# Chloride Ion Transport by the *E. coli* CLC Cl^−^/H^+^ Antiporter: A Combined Quantum-Mechanical and Molecular-Mechanical Study

**DOI:** 10.3389/fchem.2018.00062

**Published:** 2018-03-13

**Authors:** Chun-Hung Wang, Adam W. Duster, Baris O. Aydintug, MacKenzie G. Zarecki, Hai Lin

**Affiliations:** Department of Chemistry, University of Colorado Denver, Denver, CO, United States

**Keywords:** QM/MM, CLC, chloride transport, electron delocalization, conformational change, umbrella sampling, steered molecular dynamics, potential of mean force

## Abstract

We performed steered molecular dynamics (SMD) and umbrella sampling simulations of Cl^−^ ion migration through the transmembrane domain of a prototypical *E. coli* CLC Cl^−^/H^+^ antiporter by employing combined quantum-mechanical (QM) and molecular-mechanical (MM) calculations. The SMD simulations revealed interesting conformational changes of the protein. While no large-amplitude motions of the protein were observed during pore opening, the side chain rotation of the protonated external gating residue Glu148 was found to be critical for full access of the channel entrance by Cl^−^. Moving the anion into the external binding site (S_ext_) induced small-amplitude shifting of the protein backbone at the N-terminal end of helix F. As Cl^−^ traveled through the pore, rigid-body swinging motions of helix R separated it from helix D. Helix R returned to its original position once Cl^−^ exited the channel. Population analysis based on polarized wavefunction from QM/MM calculations discovered significant (up to 20%) charge loss for Cl^−^ along the ion translocation pathway inside the pore. The delocalized charge was redistributed onto the pore residues, especially the functional groups containing π bonds (e.g., the Tyr445 side chain), while the charges of the H atoms coordinating Cl^−^ changed almost negligibly. Potentials of mean force computed from umbrella sampling at the QM/MM and MM levels both displayed barriers at the same locations near the pore entrance and exit. However, the QM/MM PMF showed higher barriers (~10 kcal/mol) than the MM PMF (~2 kcal/mol). Binding energy calculations indicated that the interactions between Cl^−^ and certain pore residues were overestimated by the semi-empirical PM3 Hamiltonian and underestimated by the CHARMM36 force fields, both of which were employed in the umbrella sampling simulations. In particular, CHARMM36 underestimated binding interactions for the functional groups containing π bonds, missing the stabilizations of the Cl^−^ ion due to electron delocalization. The results suggested that it is important to explore these quantum effects for accurate descriptions of the Cl^−^ transport.

## Introduction

CLC transport proteins are a ubiquitous group of Cl^−^ ion channels and Cl^−^/H^+^ antiporters that can be found in eukaryotes and bacteria. They are associated with many critical physiological and cellular processes such as aiding extreme acid-resistance response, cell-volume regulation, and muscle contraction (Maduke et al., 2000; Dutzler, 2006; Accardi and Picollo, 2010; Jentsch, 2015). Mutations in CLC proteins cause inherited diseases in humans, such as myotonia congenita, Dent's disease, Bartter's syndrome, osteopetrosis, and idiopathic epilepsy (Jentsch, 2008). CLC transport proteins assemble and function as homodimers, of which each monomer subunit contains an independent ion translocation pore (Chen, 2005). The architecture of the transmembrane domain is highly conserved across the CLC family (Estévez et al., 2003; Lin and Chen, 2003; Chen, 2005; Engh and Maduke, 2005). While CLC channels translocate Cl^−^ ions passively, CLC antiporters mediate the *coupled* influx of Cl^−^ and the efflux of H^+^ with a 2Cl^−^/1H^+^ ratio (Accardi and Miller, 2004; Picollo and Pusch, 2005; Scheel et al., 2005; Matulef and Maduke, 2007; Miller and Nguitragool, 2009; Feng et al., 2012; Accardi, 2015).

A prototype CLC antiporter is EcCLC from *Escherichia coli*, which has been subjected to extensive structural and functional studies (Dutzler et al., 2002, 2003; Accardi and Miller, 2004; Accardi et al., 2005, 2006; Nguitragool and Miller, 2006; Walden et al., 2007; Jayaram et al., 2008; Elvington et al., 2009; Lim and Miller, 2009; Miller and Nguitragool, 2009; Picollo et al., 2009, 2012; Robertson et al., 2010; Lim et al., 2012; Vien et al., 2017). Each subunit of EcClC contains 18 α-helices, which form the ion-permeation path. In each subunit, there are three binding locations: the intracellular binding site S_int_, the central binding site S_cen_, and the extracellular binding site S_ext_. In S_int_, the Cl^−^ ion is coordinated by the backbone amine groups from Gly106 and Ser107 and may be partially hydrated (Dutzler et al., 2003; Gouaux and MacKinnon, 2005). In S_cen_, the Cl^−^ ion is coordinated by the side chain hydroxyl groups of Ser107 in helix D and of Tyr445 in helix R as well as the backbone amine groups from Ile356 and Phe357 of helix N (Dutzler et al., 2002, 2003). In the wild-type protein crystal structure, S_ext_ is occupied by the side chain of a highly conserved residue Glu148 (Dutzler et al., 2003). However, mutation of Glu148 to a charge-neutral residue results in the trapping of one Cl^−^ ion in S_ext_, where the ion coordinates with the backbone amine groups of Arg147 to Gly149 and Ile356 to Ala358 (Dutzler et al., 2003). This distinct arrangement, where the anions are extensively coordinated by the backbone amide group, is known as the “broken-helix” architecture (Dutzler et al., 2002, 2003; Feng et al., 2010). Interestingly, the charge-neutralization mutation at Glu148 converts EcCLC to a Cl^−^ channel (Dutzler et al., 2003; Accardi and Miller, 2004) and similar phenomena have been observed for mammalian ClC-4 and ClC-5 proteins (Zdebik et al., 2008). It has been suggested that the wild-type crystal structure represents the closed state and the E148Q mutation structure, the open state (Dutzler et al., 2003).

The structural information provides an important starting point for comprehending the ion binding and permeation. A number of computer modeling and simulations of CLC transport proteins have been carried out to study the mechanisms of ion transfer (Bostick and Berkowitz, 2004; Cohen and Schulten, 2004; Corry et al., 2004; Faraldo-Gómez and Roux, 2004; Miloshevsky and Jordan, 2004; Yin et al., 2004; Bisset et al., 2005; Gervasio et al., 2006; Cheng et al., 2007; Engh et al., 2007a,b; Kuang et al., 2007; Wang and Voth, 2009; Coalson and Cheng, 2010, 2011; Ko and Jo, 2010a,b; Miloshevsky et al., 2010; Kieseritzky and Knapp, 2011; Smith and Lin, 2011; Zhang and Voth, 2011; Cheng and Coalson, 2012; Picollo et al., 2012; Yu et al., 2012; Church et al., 2013; Nieto-Delgado et al., 2013; Han et al., 2014; Pezeshki et al., 2014; Chen and Beck, 2016; Jiang et al., 2016; Khantwal et al., 2016; Lee et al., 2016a,b,c; Chenal and Gunner, 2017). Regarding Cl^−^ transport, all studies agree that the protonation of the external gating residue Glu148 is key to antiporter activation and that Ser107 and Tyr445 form an internal gate. Moreover, the reported free-energy barriers for Cl^−^ translocation are relatively low, ranging from 3 to 8 kcal/mol (Cohen and Schulten, 2004; Bisset et al., 2005; Gervasio et al., 2006; Ko and Jo, 2010a; Cheng and Coalson, 2012). However, some issues are still controversial, such as whether the crystal structures of E148A and E148Q mutants correspond to the truly open state (Dutzler et al., 2003; Miloshevsky and Jordan, 2004) and how protonation of E148 is coupled to Cl^−^ binding. (Dutzler et al., 2003; Bostick and Berkowitz, 2004; Miloshevsky and Jordan, 2004; Yin et al., 2004; Gervasio et al., 2006; Ko and Jo, 2010b) The disagreements are partly due to differences in the employed methodology, constructed models, and adopted parameters. While these calculations shed light on the operating mechanisms of CLC transport, complementing experimental measurements, many molecular details are yet to be elucidated.

One long-standing puzzle concerns the extent of protein conformational change associated with Cl^−^ ions passage. The crystal structures of the protein are highly similar in both the closed and open states (Dutzler et al., 2003). This observation prompted people to propose that only local side chain motions are involved in the EcCLC operation. This mechanism differs fundamentally from other antiporters where significant motions of helixes or even domains are necessary (Feng et al., 2010, 2012). On the other hand, recent experiments on mutants that are restrained through cross linking between selected helixes suggested that rigid-body movements of certain helixes play a role (Basilio et al., 2014; Khantwal et al., 2016). Molecular dynamics (MD) simulations so far have not observed significant global conformational changes of the protein; but this could be because global rearrangements can occur in time scales longer than what the simulations have covered. On the other hand, normal-mode vibrational analysis suggested that large-amplitude swinging of helixes A and R may be one such conformational change (Miloshevsky et al., 2010). However, it is possible that local and global conformational changes both contribute to some extent.

Carrying a significant charge, Cl^−^ can strongly polarize nearby solvent molecules and pore residues. Moreover, under certain circumstances, the excess charge of Cl^−^ can easily delocalize to the ion's surroundings. Previous quantum-mechanical (QM) calculations using truncated models of EcCLC have indeed revealed substantial mutual polarization and partial charge transfer for the Cl^−^ ions in the binding sites (Smith and Lin, 2011; Church et al., 2013; Nieto-Delgado et al., 2013). The most prominent manifestations of polarization and charge transfer were found among the π orbitals of the nearby protein atoms, e.g., the atoms of the backbone peptide links and of the side chains of Glu148 and Tyr445. Furthermore, energy decomposition analysis confirmed that the quantum effects of electron delocalization over Cl^−^ and the π-orbitals atoms contributed significantly to the stabilization of the Cl^−^ ions in the biding sites (Church et al., 2013). On the other hand, it has been demonstrated that these induction effects can impact the ion binding and translocation in cation channels (Compoint et al., 2004; Allen et al., 2006; Huetz et al., 2006; Bucher et al., 2007, 2010; Dudev and Lim, 2009; Illingworth and Domene, 2009; Bostick and Brooks, 2010; Illingworth et al., 2010; Varma and Rempe, 2010; Roux et al., 2011; Wang et al., 2012). Do the extensive charge redistributions of Cl^−^ affect its transport in EcCLC? It will be interesting to find out.

In this study, we investigate possible protein conformational changes and explore how electron delocalization affects the ion's expedition through EcCLC. We carry out dynamics simulations at both the molecular mechanical (MM) and the combined quantum-mechanical/molecular-mechanical (QM/MM) levels (Warshel and Levitt, 1976; Field et al., 1990; Gao, 1996; Zhang et al., 1999; Rode et al., 2006; Lin and Truhlar, 2007; Senn and Thiel, 2007; van der Kamp and Mulholland, 2013; Pezeshki and Lin, 2015; Duster et al., 2017). We perform steered molecular dynamics (SMD) simulations (Izrailev et al., 1998) to escort a Cl^−^ ion through the pore. We examine the changes in pore size and protein conformations as the ion moves through and estimate the associated potential of mean force (PMF) using umbrella sampling (Torrie and Valleau, 1977; Roux, 1995). By comparing and combing the MM and QM/MM results, this study will deepen our understanding of the Cl^−^ translocation process operated by EcCLC.

## Computational details

### Model preparation

The model system was constructed based on the crystal structure of the wild-type (WT) protein's transmembrane domain (PDB code: 1OTS) (Dutzler et al., 2003). Because the two subunits each function independently, only one subunit (chain A) was used to build the model system. The protonation states of the residues were set according to an earlier Poisson-Boltzmann calculation (Faraldo-Gómez and Roux, 2004) except for the external gate Glu148, which was protonated. The protonated Glu148 was manually rotated outwards to open the pore. Therefore, we were simulating the open state of the protein. The protein with the two bound Cl^−^ ions in the S_int_ and S_cen_ sites was embedded in a two-layer 1-palmitoyl-2-oleoyl-sn-glycero-3-phosphoethanolamine (POPE) lipid bilayer. The protein-membrane complex was then solvated by adding water molecules on both sides. The thickness of the slab of water at either side was about 20 Å. We then replaced 109 randomly selected water molecules by 58 K^+^ and 51 Cl^−^ ions to neutralize the total charge and to achieve an approximate 0.15 M physiological salt concentration for the solution. The primary cell of the model system consists of 6823 protein atoms, 18308 water molecules, 60 Cl^−^ ions, 51 K^+^ ions, and 292 POPE molecules. The protein, lipid, and ions were described by the CHARMM36 force fields (MacKerell et al., 1998; Mackerell et al., 2004; Klauda et al., 2010; Best et al., 2012), and water by the TIP3P model (Jorgensen et al., 1983).

The system was equilibrated under the *NpT* ensemble at *p* = 1 bar and *T* = 310 K for 10 ns, followed by the *NVT* ensemble at *T* = 310 K for 45 ns, with final unit cell dimensions of 101.3 × 99.0 × 92.9 Å^3^. Temperature was controlled through Langevin dynamics (Phillips et al., 2005) where the temperature dampening coefficient was set to 1.0 ps^−1^, and pressure through Langevin piston Nosè-Hoover method (Martyna et al., 1994; Feller et al., 1995) with a barostat oscillation period of 175 fs and a damping time of 150 fs. Periodic boundary conditions were employed, and long-range electrostatic interactions were computed by particle mesh Ewald (PME) method (Darden et al., 1993; Essmann et al., 1995). A 14.0 Å cutoff was used for nonbonded interactions, with smoothing switch at 13.0 Å and pair lists cutoff at 16.0 Å. The SHAKE algorithm (Ryckaert et al., 1977) was used to make waters rigid as well as to constrain all bonds between hydrogen and heavy atoms. A time step of 2 fs was used. The equilibrations were performed by using the NAMD (Phillips et al., 2005) program version 2.10. The saved trajectories were visually inspected using the program VMD (Humphrey et al., 1996).

### Steered molecular dynamics

The Cl^−^ transport by EcCLC is complicated due to the coupling with the H^+^ migration, and a 2Cl^−^/1H^+^ ratio has been established (Accardi and Miller, 2004; Picollo and Pusch, 2005; Scheel et al., 2005; Matulef and Maduke, 2007; Miller and Nguitragool, 2009; Feng et al., 2012; Accardi, 2015). However, exactly how the two Cl^−^ ions were transferred in every cycle is still under debate. As such, we only considered the uncoupled translocation of one Cl^−^ ion to simplify the analysis, which suffices the purpose of this work.

**Figure 1:**
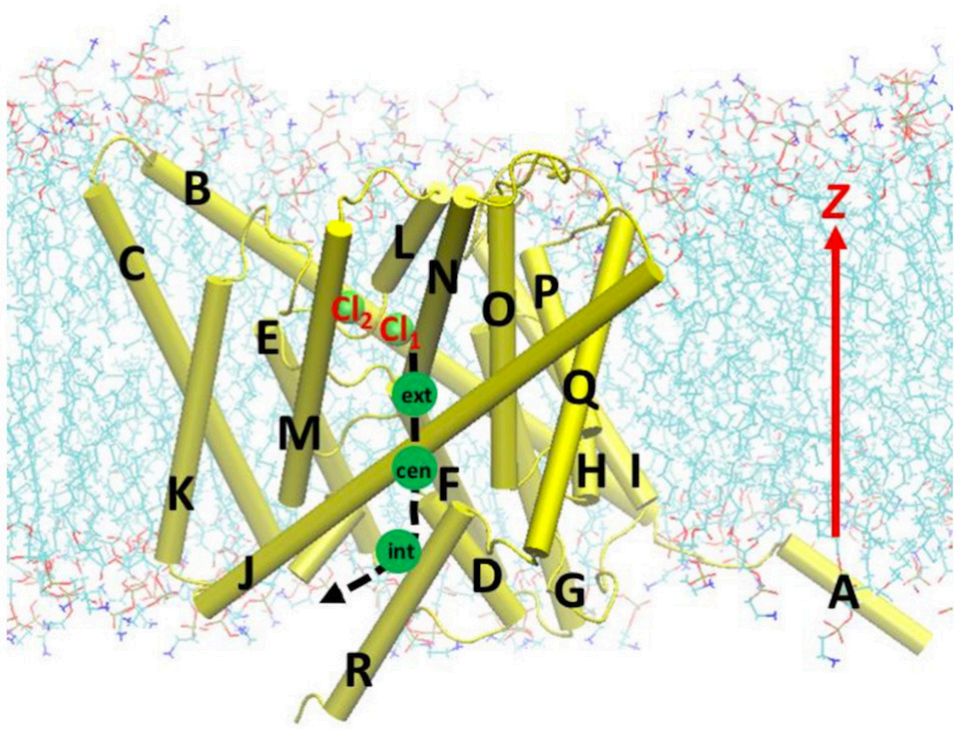
Beginning geometry for steered molecular dynamics simulations, where the Cl^−^ ion at the extracellular pore entrance (Cl_1_) was moved toward the intracellular side along the path indicated by the black dash curve and arrow. Approximate positions of the three binding sites S_ext_, S_cen_, and S_int_ were indicated by the green circles. Also shown is another nearby Cl^−^ ion (Cl_2_), which was attracted to Arg147 during the equilibration. The EcCLC antiporter transmembrane domain was illustrated in cartoon in yellow, with the helixes A–R labeled. Lipid molecules were depicted as lines (cyan, C; blue, N; red, O; and tan, P). For clarity, water molecules and ions are not displayed, except for the above two Cl^−^ ion shown as green spheres.

The parameters and program used in the model system equilibration were used in the constant-velocity SMD (Izrailev et al., 1998) simulations, except those indicated below. Before the SMD simulations, the Cl^−^ ion at S_cen_ in the equilibrated model was replaced and fixed at the extracellular side at *z* = 0, and the model system was re-equilibrated for 2 ns. The resulting geometry, where the Cl^−^ ion stayed just outside the extracellular pore entrance, served as the starting geometry for the SMD simulation (see Figure 1). For the SMD simulations, the Cl^−^ ion was dragged through the pore toward the intracellular side from *z* = 0.0 to −20.0 Å. Three steering speeds were tested: 2, 5, and 10 Å/ns. (As a comparison, the slowest speed applied in a previous study, Ko and Jo, 2010a was 10 Å/ns). The steering forces were applied along the –*z* direction, and the spring force constants were set to 10 kcal/mol/Å^2^. The trajectories were saved every 100 steps. The Cα atoms of randomly selected protein residues Gly141, Thr226, Ala325, and Leu421, which belongs to helixes E, I, L, and Q, respectively, were restrained to their initial positions by harmonic potentials with force constants of 1.0 kcal/mol/Å^2^ to prevent the protein from translating with the Cl^−^ ion. The simulations were conducted with constant volume and constant temperature. The time step was set to 1.0 fs, as the SHAKE algorithm was not used (the same applied to umbrella sampling below).

### Umbrella sampling

The starting geometries for umbrella sampling calculations were extracted from the trajectory of the SMD simulations at the slowest speed of 2 Å/ns. The *z* component of the Cl^−^ ion's coordinate was chosen to be the reaction coordinate. In total, there were 41 sampling windows covering 0 > *z* > −20.0 Å, with equal spacing of 0.5 Å and each window containing the Cl^−^ ion initially located approximately at the window center. The simulations were done at both the QM/MM and MM levels. The PMF was obtained using the weighted histogram analysis method (WHAM) (Kumar et al., 1992).

**Figure 2:**
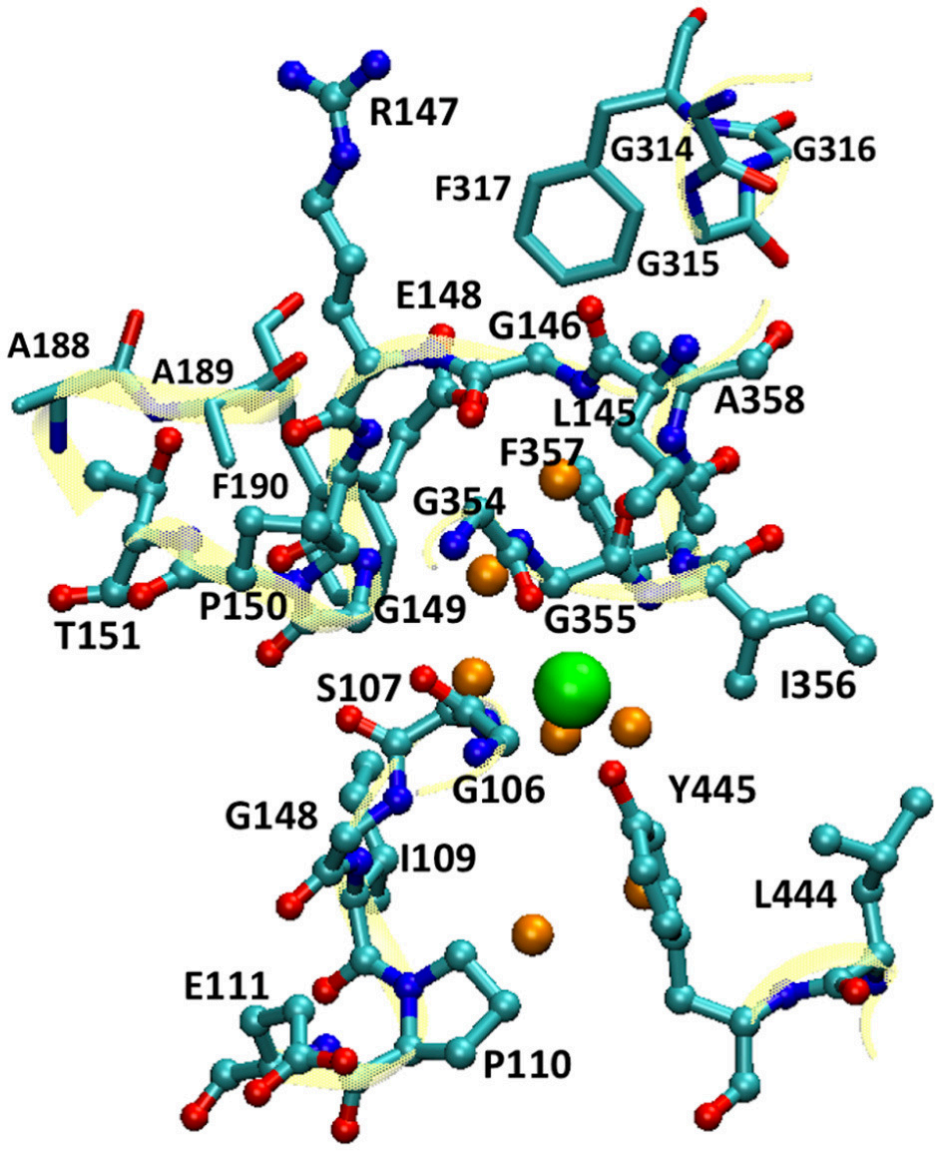
The QM subsystem in QM/MM umbrella sampling simulations. The pore residues are shown as balls and sticks, and the residues near the extracellular pore entrance as sticks (cyan, C; blue N; and red O). The backbones of these residues are illustrated in cartoon in yellow. The Cl^−^ ion is depicted as a large green sphere, and the water molecules in its vicinity as small orange spheres. For clarity, H atoms are omitted.

For the QM/MM umbrella sampling, the QM subsystem covered the entire pore section that the Cl^−^ ion traverses. This included the Cl^−^ ion, the pore residues (Gly106 to Pro110, Glu111 backbone, Leu145 to Pro150, Thr151 backbone, Gly354 to Ala358, Leu444, and Tyr445), selected residues near the extracellular pore entrance (Ala188 to Phe190 and Gly314 to Gly317), and 7 water molecules in the vicinity of the Cl^−^ ion (Figure 2). For simplicity, the mechanical-embedding scheme with H as link-atom was adopted (Bakowies and Thiel, 1996; Lin and Truhlar, 2005). For the first and last residues in a list of consecutive residues in the QM subsystem, the amine and carbonyl groups were replaced by H atoms, respectively. When the QM/MM boundary separated backbone and side chain, H atoms were used to cap the Cα (or Cβ) atom. To prevent MM water molecules from diffusing into the QM region, the MM water molecules adjacent to the QM subsystem (within 10.0 Å from the QM subsystem) were restrained by imposing harmonic potentials to their O atoms with force constants of 1.0 kcal/mol/Å^2^. The QM subsystem thus covered the entire pore section that the Cl^−^ ion passed through. For computational efficiency, the PM3 (Stewart, 1989a,b) semi-empirical Hamiltonian was chosen to model the QM subsystem. Higher-level electronic-structure methods are more accurate, but they were not employed for umbrella sampling due to their much higher computational costs. Periodic boundary conditions with a minimum-image convention were employed, where the cutoffs for electrostatic and van der Waals interactions were set to 14.0 Å with smoothing switches at 13.0 Å. For each window, equilibration of 10 ps and production of 200 ps were performed. A Nose-Hoover thermostat (Hoover, 1985; Kreis et al., 2016) with a coupling constant of 4.0 fs was applied to control the temperature. The force constants of the bias harmonic potential were set to 14 kcal/mol/Å^2^. The QM/MM simulations were done using a local version of the QMMM program (Lin et al., 2017), which called the MNDO program (Thiel, 2005) for QM calculations and NAMD for MM calculations, synthesized the QM and MM gradients, and propagated the trajectory.

For the MM umbrella sampling, longer simulations could be afforded, and 0.5 ns equilibration followed by 2.0 ns production run was performed for each window. The force constants of the bias harmonic potential were set to 5 kcal/mol/Å^2^. The same setups and program used in the model system equilibration were again used here, unless otherwise noted.

### Pore radius calculations

The pore radius at the Cl^−^ ion locations were calculated for representative geometries extracted from umbrella sampling simulations. This analysis was carried out for both the MM and QM/MM simulations. In either case, 7 snapshots were extracted from the production phase of the trajectory for each of the 41 windows. The pore radii were computed utilizing the program HOLE (Smart et al., 1996), where a Monte Carlo-based search algorithm was employed to identify the best route for a sphere with a given radius to pass through the protein. The “hard-core” radii in Turano et al. (1992) was adopted for the calculations, which implicitly account for the thermal motions of atoms. The results of the 7 representative geometries were then averaged for a given window.

### Electron delocalization

To characterize the Cl^−^ electron delocalization during the ion translocation along the pore, electrostatic-embedding QM/MM single-point calculations were carried out on one representative geometry for each window of QM/MM umbrella sampling. The QM subsystem was the same as the one in the umbrella sampling except that the selected residues near the pore entrance (Ala188 to Phe190 and Gly314 to Gly317) were excluded; these residues were treated by MM due to computational cost considerations. We employed the charge-redistribution scheme for the boundary treatment (Lin and Truhlar, 2005). The B3LYP (Becke, 1988, 1993; Lee et al., 1988) functional with the 6-31+G(d) basis set (Ditchfield et al., 1971; Hehre et al., 1972; Francl et al., 1982; Clark et al., 1983) were used for the QM description, and the MM force fields were the same as those in the umbrella sampling. All MM atoms within 12.0 Å from the QM subsystem were included as background point charges in the embedded-QM calculations. The calculations were performed using the local version of QMMM, which called *Gaussian09* (Frisch et al., 2010) for the QM calculations. The Löwdin charges (Löwdin, 1950; Wang et al., 2013) and the natural charges based on Natural Bond Orbital analysis (Foster and Weinhold, 1980; Reed et al., 1985) were computed using the polarized electron density of the QM subsystem. We tested and found that further inclusion of more MM background point charges in the embedded-QM Hamiltonian did not noticeably change the atomic charges for the QM atoms.

## Results

### Equilibrated model system

During equilibration, the protonated Glu148 side chain, which had been manually rotated out of S_ext_, moved toward S_ext_ and partially obstructed the pore entrance, but it did not enter the pore to reclaim the binding site. Located at the transition area between the intracellular aqueous solution and the pore, the Cl^−^ ion at S_int_ drifted away during the equilibration. This is in line with the weak binding affinity of Cl^−^ at this site (Lobet and Dutzler, 2006), suggesting that S_int_ is less critical than S_ext_ and S_cen_ to the Cl^−^ transport. Attracted by the positively charged Arg147, Cl^−^ ions from the bulk often diffused to near the pore entrance on the extracellular side and stayed there for up to 1 ns. This additional weak binding site has been reported in previous computational studies, although its exact location varied in the literature (Bostick and Berkowitz, 2004; Cohen and Schulten, 2004; Faraldo-Gómez and Roux, 2004; Yin et al., 2004; Gervasio et al., 2006; Smith and Lin, 2011; Church et al., 2013). It is quite possible that this additional binding site plays a role in the Cl^−^ ion recruitment.

### Conformational changes along Cl^−^ translocation

Our analysis on the protein conformational changes will put emphasis on the SMD trajectories, where a complete and continuous voyage was simulated for the Cl^−^ ion through the pore.

#### Global conformational changes of the protein

Generally speaking, the SMD trajectories of the three tested steering speeds showed rather similar changes in the protein conformation, but the variations became more prominent as the steering speed decreased. The overall conformational changes can be quantified by root-mean-square-deviation (RMSD) values of the protein backbones, which are plotted as functions of the simulation progress (Figure S1). All trajectories show a trend of increasing RMSD over time. While the trend was moderate at the speeds of 5 and 10 Å/ns, it was considerably enhanced at 2 Å/ns. While this could be incidental, we suspect that it is at least partly due to the longer relaxation time permitted for the model system at a slower steering speed. As such, we will present and discuss the data obtained with the slowest speed of 2 Å/ns, while bringing in the results of 5 and 10 Å/ns only when necessary.

**Figure 3:**
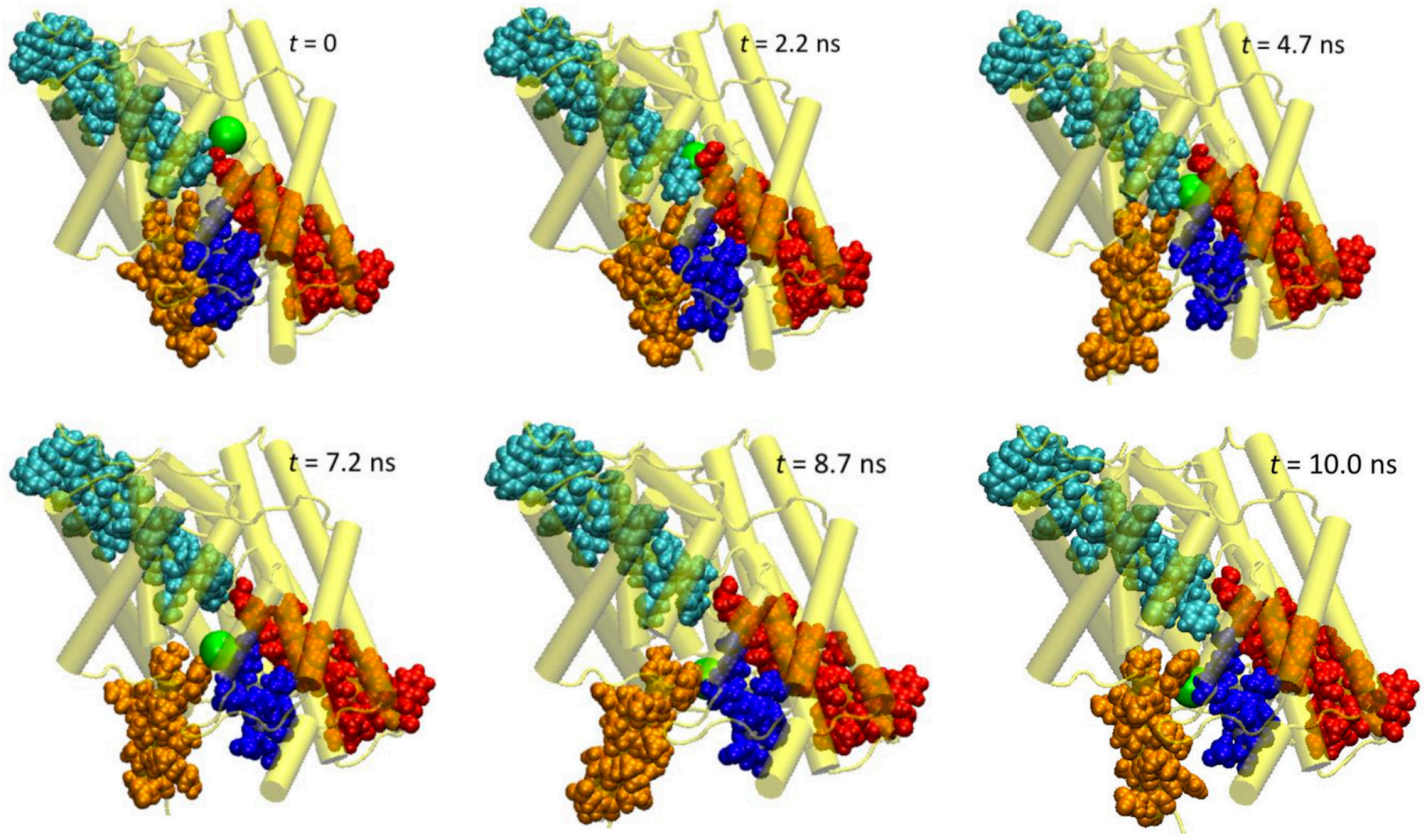
Extracted snapshots from the trajectories of the steered molecular dynamics simulations at a steering speed of 2 Å/ns. The protein is shown as cartoon in yellow, helixes D (blue), F (red), N (cyan), and R (orange) as space-filled van der Waals spheres, and the Cl^−^ ion as a large green sphere.

**Figure 4:**
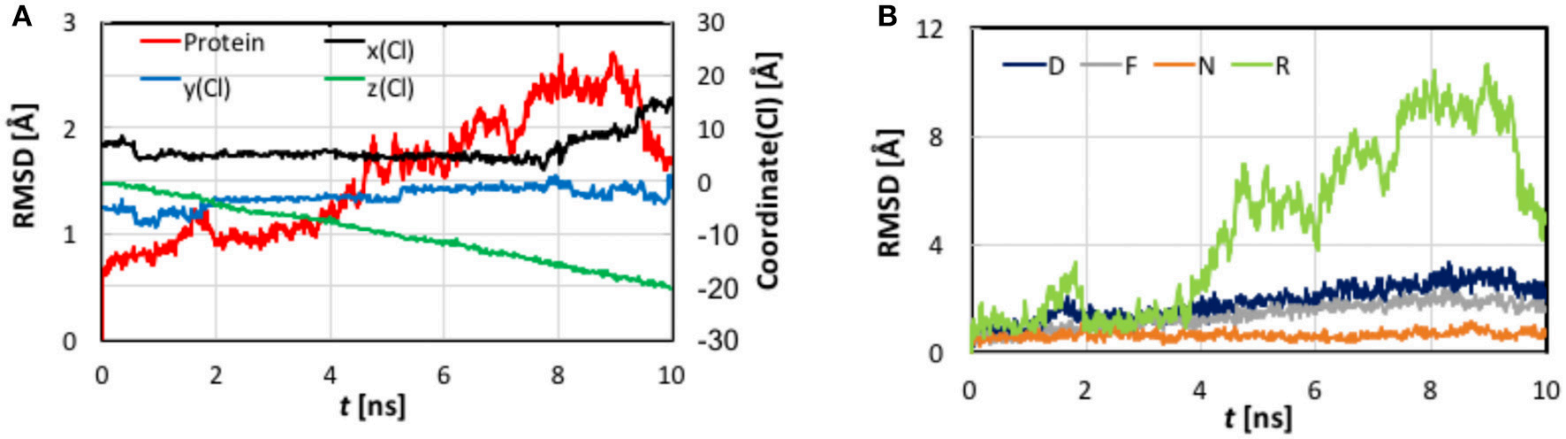
**(A)** Cartesian coordinates of the transferring Cl^−^ ion and root-mean-square-deviation (RMSD) values of the protein backbone heavy atoms as functions of the simulation time of the steered molecular dynamics simulations with a steering speed of 2 Å/ns. **(B)** The RMSD values of the helixes D, F, N, and R, which form the pore.

First, let us look at the global backbone movements induced by the Cl^−^ passage. Figure 3 shows the orientations of the four helixes (D, F, N, and R) that define the pore in selected snapshots extracted from the SMD trajectory. In Figure 4, we display, vs. the simulation time *t*, the Cartesian coordinates of the Cl^−^ ion as well as the backbone RMSD values of the protein and of these four helixes. (See Figure S2 for the backbone RMSD values of the complete set of all helixes).

As evident from Figure 4, the *z* coordinate of the Cl^−^ ion varied linearly as a result of constant-velocity steering. The journey of the ion can be divided into three stages. In the first 2 ns, which we call the “entrance” stage, the ion wandered around in the extracellular vestibule just outside the entrance (*z* ~ −4 Å) before finding its way into the pore. In the second “central” stage of *t* = 2–8 ns, the ion walked essentially straight down the path, as manifested by the approximately constant *x* and *y* coordinates. During this section, the ion traveled through S_ext_ at *z* ~ −6 Å and S_cen_ at *z* ~ −10 Å. The ion's arrival at the kink (*z* ~ −16 Å) at *t* = ~8 ns marked the beginning of the last “exit” stage. The deviation in the *y* coordinate indicated that the pore is curved along this part. On its way out, the ion passed S_int_ (*z* ~ −18 Å) at *t* = ~9 ns.

The protein backbone RMSD increased slowly in the first 4 ns, implying that there were relatively small changes in the protein conformation during the entrance and early-central stages. The RMSD then grew rapidly in the next 4 ns from ~1 Å to ~2.5 Å, remained there for ~1 ns, and dropped sharply down to 1.5 Å in the last 1 ns. The substantial rise and fall in the RMSD in 4–10 ns indicated significant conformational changes of the protein over this period. The conclusion is exemplified by the representative snapshots in Figure 3, where large-amplitude rigid-body displacements of part of the protein can be seen during this time. Three helixes were identified in the large-amplitude rigid-body displacements induced by the Cl^−^ penetration: helix R most prominently and helixes D and F moderately, all of which are critical to the ion passage (Figure 4B).

- *Helix R*: This helix, which contains the internal gating residue Tyr445, exhibited marked swinging motions similar to a door opening and closing. This motion corresponds with a normal mode presented in an earlier study (Miloshevsky et al., 2010). It appeared that the movement of Tyr445 propagated through the helix, giving rise to the observed big changes in the backbone RMSD. Separating helixes R and D, the outward swinging reached its maximum at ~8.7 ns. After that, the ion, which had been following the tilted path since *t* ~ 7.9 ns, exited the pore, and helixes R and D returned to their original positions (though not completely restored in the next 2 ns). Because helix R extrudes into the intracellular solution, it is less restrained than the other helixes by the rest of the protein or by the membrane, thus possessing larger flexibility. However, one limitation of the current model is that it contains only one subunit. In the dimer structure, helix R may interact with helix A from the other subunit. Moreover, the C-terminal domain, which was not included in this model, is connected to helix R and may also reduce its mobility. Therefore, the motion amplitude of helix R was likely exaggerated here.
- *Helix D*: Another important residue along the Cl^−^ pathway is Ser107, which is situated at the beginning of helix D and, together with Y445, contributes to Cl^−^ binding at S_cen_. The movement of the Ser107 also propagated through helix D and contributed to changes in the backbone RMSD. However, the shorter helix D is embedded deeper into the protein matrix and thus constrained more than helix R. Unsurprisingly, helix D moved only moderately.
- *Helix F*: The S_ext_ site is located between the ends of helixes F and N, where the amine groups of the backbone extensively coordinate ion. A bottleneck exists between S_ext_ and the pore entrance at the extracellular vestibule. However, it seemed that neither helix F nor N underwent significant global rigid-body movements when the Cl^−^ ion passed the bottleneck, implying that the adaptations to accommodate the ion's passage were primarily local. Similar conclusions were drawn in an earlier SMD simulations at a higher steering speed of 10 Å/ns (Ko and Jo, 2010a). During the late central and exit stages of the Cl^−^ excursion (*t* > 4 ns), helix F displayed swinging motions, albeit only to a modest extent, probably because it was pushed by the nearby helix D in response to the internal gate's opening/closing.

We note that helix A backbone RMSD also exhibited significant changes. Similar to helix R, helix A was exposed to the solvent and thus was quite mobile. However, the trend of its backbone RMSD change was not consistent across the three simulations with various steering speeds (Figure S3). Because helix A resides far from the rest of the protein in the current model, its motions were largely independent of the excursion of the Cl^−^ ion in this study.

A small “bump” in the backbone RMSD of the protein from *t* = 1–2 ns was predominantly due to the small changes in the backbone RMSD of helixes D and R. Inspection of the trajectory unveiled that this was caused by the highly flexible Phe357 side chain, the fluctuation of which disturbed Tyr445 and, in turn, Ser107. However, this happened when the Cl^−^ ion was still in the extracellular vestibule, and Tyr445 had roughly returned to its initial position by *t* = 2 ns when the ion began entering the pore. Moreover, such a bump was not observed in the simulations of 5 or 10 Å/ns speed. Therefore, this bump seemed irrelevant or insignificant to Cl^−^ transport.

The protein conformations featured in the SMD trajectories were preserved in the trajectories generated by both the QM/MM and MM umbrella sampling simulations. Because the MM umbrella sampling allowed even longer equilibration (2.5 ns for each window), the observation of similar conformations in both the SMD and umbrella sampling simulations supports the hypothesis that other major conformational changes besides the helix R movement, if any, should occur at longer time scales.

#### Local conformational changes of pore residues

**Figure 5:**
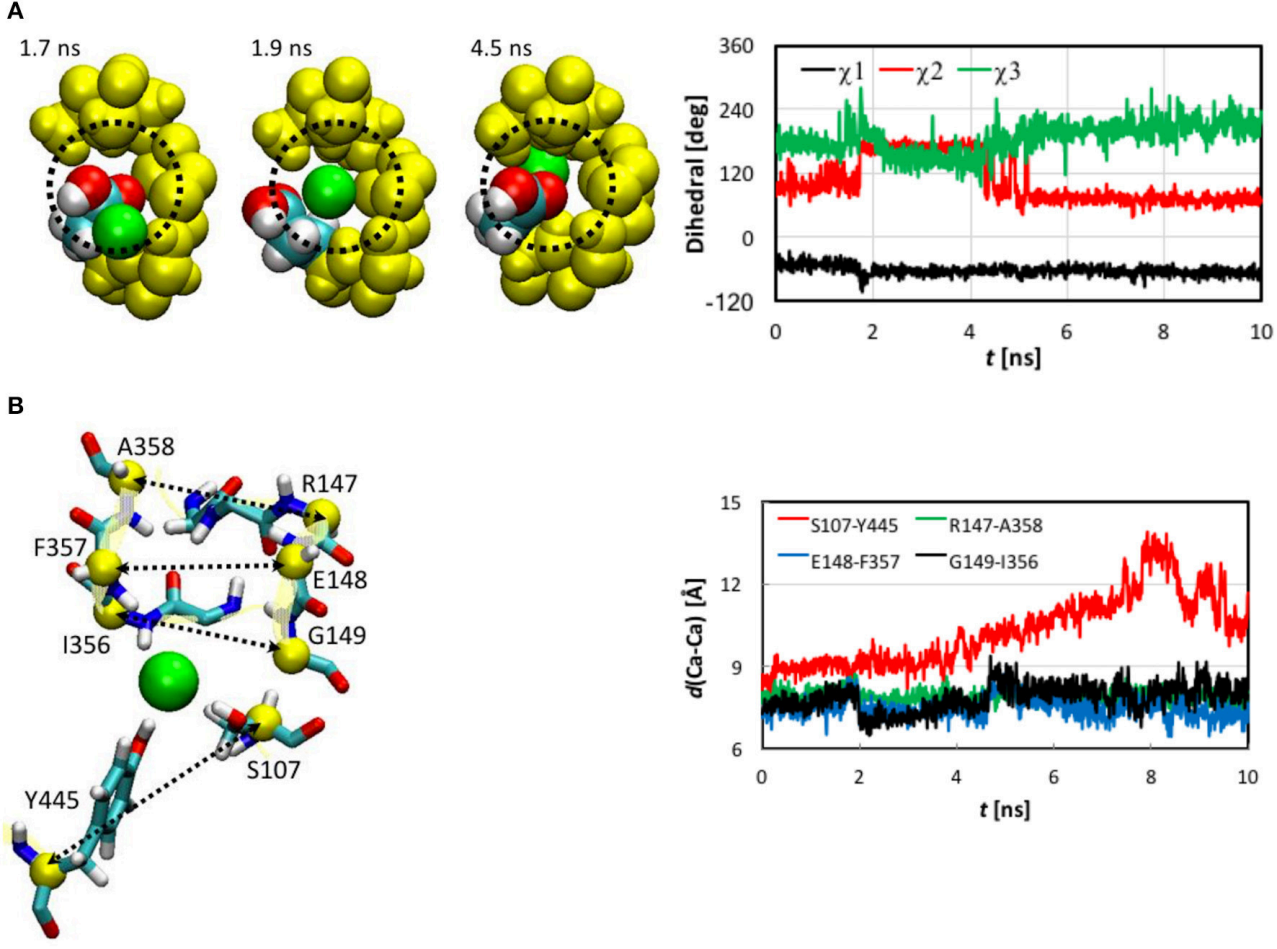
**(A)** Opening of pore entrance by flipping the Glu148 side chain. Left: Three snapshots (top view) at *t* = 1.7, 1.9, and 4.5 ns, where the Cl^−^ ion (green), Glu148 side chain (white, H; cyan, C; and red, O), backbones of 146–148 and 355–358 (yellow), and the methylene bridge (-CH_2_-) group of Phe357 (yellow) are displayed as space-filled van der Waals spheres. The dotted circle indicates the pore. Right: Dihedrals χ1-χ3 characterizing the conformations of the Glu148 side chain as functions of simulation time. Note that χ3 was shifted to be in the range of 0 to 360° instead of the standard −180° to +180° for better visualization. **(B)** Distances between pairs of Cα atoms (yellow spheres) for selected residues (yellow ribbons). Left: Drawn as sticks are Ser107, Ile356 to Ala35 (backbone only), Arg147 to Gly149 (backbone only), and Tyr445. The Cl^−^ ion is illustrated as a green sphere. Right: Distances as functions of simulation time.

Next, we examined the conformational changes of individual residues. The first thing we looked at was how the Cl^−^ ion entered the pore. In the entrance stage of its voyage, the Cl^−^ ion was accompanied by the side chain carbonyl group of Glu148 as well as by the backbone amine groups of Arg147 and Glu148 (Figure S5). Inspection of the trajectory discovered that at *t*~1.8 ns the side chain carboxyl group of Glu148, which had obstructed the pore entrance, flipped, revealing the pore for the Cl^−^ ion to access. The side chain returned to its initial orientation after the ion passed through S_ext_. The conformational change of Glu148 side chain was characterized by a 60° rotation of dihedral χ_2_, while χ_1_ and χ_3_ remained largely unchanged (Figure 5A).

The Cl^−^ ion in S_ext_ was held by the backbone amine groups of Gly147 to Gly149 and Ile356 to Ala358 (Figure 5B). The Gly146 backbone also stayed close, but the minimum distance from its amine H to the Cl^−^ was 3.3 Å, much longer than the other residue backbones considered here, for which the distances could reach 2.1 Å or shorter. We thus conclude that this interaction was not so critical as the other residue backbones. As the Cl^−^ ion entered S_ext_, the backbones of Glu148 and Gly149 from helix F shifted closer to Cl^−^ to better solvate the ion. In contrast, the backbones of Ile356 to Ala358 from helix N displaced less significantly, which can again be attributed to the tighter embrace of the N helix by the protein matrix. These local conformational changes were well captured by the variations in the backbone RMSD of these residues (Figure S4). Rapid increases can be seen in the backbone RMSD of Glu148 and Gly149 at ~2.0 ns. (Note that the side chain RMSD of Glu148 increased ~0.2 ns earlier than the rise in the backbone RMSD of Glu148 due to pore opening). The backbone RMSD values of Glu148 and Gly149 remained high for ~2.4 ns before appreciably reducing. This period of ~2.4 ns corresponded to the ion cruising through S_ext_. Interestingly, although the Phe357 side chain was highly mobile all the time, its activities did not seem very relevant.

The shifts of Glu148 and Gly149 backbones were also reflected by the distances between the Cα atoms of the involved residues (Figure 5B), where distances reduced by ~1 Å were observed for the Glu148-Phe357 and Gly149-Ile356 pairs near *t* = 2.0 ns and remained low for ~2.4 ns. However, we note that, unlike the flipping of Glu148 side chain, the shifts of Glu148 and Gly149 backbones were not a prerequisite of the Cl^−^ ion passage through S_ext_. Rather, they were a consequence. In other words, the ion could still have cruised through without the movement of the Glu148 and Gly149 backbones, because the pore sizes were large enough in this section. In fact, it has been known that even bigger anions such as Br^−^ can also be transported rather efficiently (the relative permeability $P_{\text{Br}}^{-}$/$P_{\text{Cl}}^{-}$ = 0.7; White and Miller, 1981; Gouaux and MacKinnon, 2005). The lack of high selectivity between Cl^−^ and Br^−^ is probably not problematic physiologically because of the much lower physiological abundance of Br^−^ than Cl^−^ ions (Gouaux and MacKinnon, 2005).

The Ser107 and Tyr445 side chains played a central role in the late central and exit stages of the Cl^−^ ion's expedition. The side chain hydroxyl groups of Ser107 and Tyr445 accommodated the Cl^−^ ion as soon as it left S_ext_ (*t* ~ 4.4 ns) and escorted it all the way through its exit from the channel. Maintaining the ion coordination by these two residues drove the movement of helixes D and R and led to the temporary separation of the two helixes. Consequently, the distance between the Cα atoms of Ser107 and Tyr445 increased until the ion exited from the pore (Figure 5B). Interestingly, we did not observe much rotation of the Ser107 side chain after 4.4 ns, during which χ1 was mostly constant (Figure S6). The attraction by the Cl^−^ ion might have somewhat reduced the mobility of this side chain. The Tyr445 side chain χ1 and χ2 did not really change throughout the entire journey. The side chain hydroxyl groups of both residues remained quite flexible, though.

#### Pore sizes

**Figure 6:**
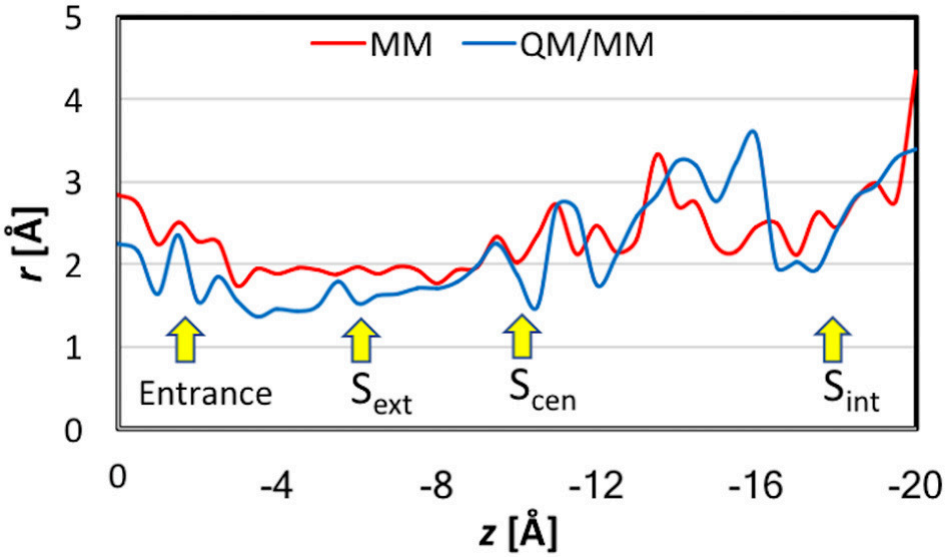
Pore sizes at the locations of the Cl^−^ ion in the umbrella sampling simulations. For each location, the reported radius is the average over seven representative geometries.

Figure 6 displays the pore sizes at the locations of the Cl^−^ ion in the umbrella sampling simulations. For each location, the radius was averaged over 7 representative geometries. Please note that this is different from the pore sizes along the pathway for a given snapshot of the protein. The pore size plot here reflects the binding environment of the Cl^−^ ion as it travels down the channel. Nevertheless, the overall shapes of the MM and QM/MM pore-size curves agree reasonably well with those reported previously (Bostick and Berkowitz, 2004; Corry et al., 2004; Yin et al., 2004; Kuang et al., 2008; Ko and Jo, 2010a; Khantwal et al., 2016). The MM pore radius was in general larger by up to 0.5 Å than the QM/MM radius, especially at the binding sites.

### Cl^−^ ion charge redistribution

**Figure 7:**
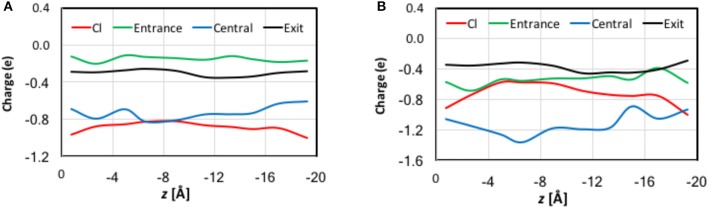
QM charges for the Cl^−^ ion and the total charges for the entrance, central, and exit cohorts of residue functional groups against the reaction coordinate *z* for representative geometries in QM/MM umbrella sampling simulations. Note that to assist better visualization, we have divided the reaction pathway into 10 sections (each of 2 Å long) and collected the average charges over each section. See the text for the cohort definitions. **(A)** Natural charges. **(B)** Löwdin charges.

In previous studies, (Smith and Lin, 2011; Church et al., 2013; Nieto-Delgado et al., 2013) it was found that the charge carried by the Cl^−^ ion is delocalized to its solvation shell when it is bound at S_ext_ and S_cen_. In the present work, we examined the trend along the entire pore. The QM charges were computed using the electrostatic-embedding QM/MM scheme on representative geometries from umbrella sampling simulations. Compared with our early truncated-QM model calculations for the pore in the gas phase (Smith and Lin, 2011; Church et al., 2013) here the electrostatic-embedding QM/MM calculations more realistically incorporated the polarization effects due to the protein/solvent environment. The results for the Cl^−^ ion and for selected atoms and functional groups belonged to three residue cohorts (described later in this section) are depicted in Figure S7 as functions of the reaction coordination *z*. The total charges of each cohort are displayed in Figure 7. To better assist visualization, we have divided the reaction pathway into 10 sections (each of 2 Å long) and collected the average charges over each section. Importantly, both the natural and Löwdin charges gave qualitatively similar descriptions, although the Löwdin charges probably exaggerated the extents of charge variation. Therefore, our analysis will focus on the natural charges.

The functional groups in a given cohort were those who critically participated in the Cl^−^ coordination at the corresponding stage of ion transport.

- *Entrance cohort*: side chain carboxyl group of Glu148 and backbones of Arg147 and Glu148
- *Central cohort*: side chain hydroxyl group of Ser107, side chain of Tyr445, and backbones of Arg147–Gly149 and Ile356–Ala358
- *Exit cohort*: side chain hydroxyl group of Ser107 and side chain of Tyr445

Note that there are overlaps in the assignment of the functional groups, because one functional group might participate in the Cl^−^ ion solvation in more than one stage. For example, the entire exit cohort was part of the central cohort. Because our earlier QM analyses had discovered that the transferred charge would be delocalized over all nearby atoms with π bonds (Church et al., 2013) we treated the entire backbone of a residue rather than just the amine group as one unit in the present analysis. The same was applied to the ring in the Tyr445 side chain.

There are significant charge redistributions between Cl^−^ and the coordinating residues along the ion transport pathway. In the external vestibules, if the Cl^−^ ion had no contact with any cohort residues, it retained a charge of nearly −1.0 e, despite being coordinated by water molecules. As soon as it interacted with the entrance cohort, notable partial charge transfer was observed. The amount of charge loss increased as the ion advanced through the pore and interacted with more residues. The maximal charge deduction was ~20% and occurred when the ion traveled from S_ext_ (−6 Å) to S_cen_ (−10 Å). This is not surprising, because the central cohort has the largest number of coordinating functional groups and caused most the significant charge redistribution. Correspondingly, a dip appeared near *z* = −8 Å in the total-charge curve of the central cohort. Finally, the exit cohort withdrew some of the charge density from Cl^−^ as the ion approached it. The charge transfer to the exit cohort was the greatest from S_cen_ to the kink (−10 > *z* > −16 Å). After leaving the kink, the ion gradually regained its charge on the way out the channel. Interestingly, the H atoms that directly coordinated the Cl^−^ ion displayed almost negligible changes in their charges.

### Potential of mean force through umbrella sampling

**Figure 8:**
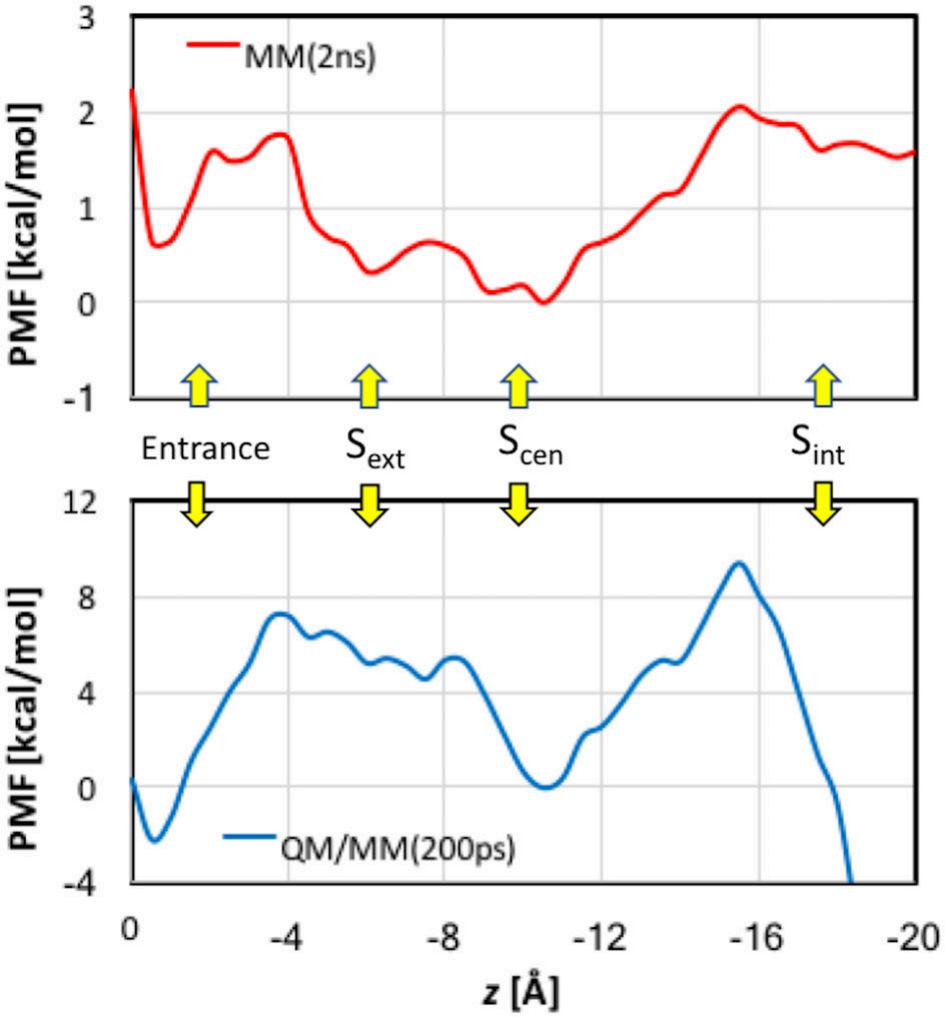
Potentials of mean force for Cl^−^ ion passage obtained by umbrella sampling simulations. Note the different scales in the MM and QM/MM plots.

When analyzing the one-dimensional PMFs, we focused on the data in the range of *z* > −16 Å, because the pore is wide and considerably tilted after *z* < −16 Å, for which multi-dimensional PMFs shall provide better descriptions of the ion dynamics. First, we checked the convergence of the MM PMF with respect to the sampling time per window (Figure S8A). The MM PMF converged to within 0.2 kcal/mol from 1.5 ns/window to 2.0 ns/window, suggesting that the sampling time length was adequate. The final MM PMF is shown in Figure 8 as the red curve. The lowest free energy in the entire PMF corresponded to S_cen_, but S_ext_ is higher by only ~0.5 kcal/mol. The well at the entrance was roughly the same as S_ext_. The PMF possesses a barrier of ~1.5 kcal/mol between the pore entrance and S_ext_. This was primarily due to the need to rotate the Glu148 side chain that blocked the channel so that the pore could be accessed. Because our model was constructed in the open state instead of the closed state, this barrier did not account for the changes needed to prepare the protein in the open state. Going from S_ext_ to S_cen_ experienced only a low barrier of ~0.3 kcal/mol. A barrier of ~2 kcal/mol must be overcome when the ion departed from the channel. This was mainly caused by the internal gate opening by Tyr445 and Ser107, the movements of which propagated to the backbones of helixes R and D.

The convergence of QM/MM PMF was less satisfactory because of the shorter sampling time (Figure S8B). Although the QM/MM PMF is probably not fully converged, the shape of the curve was distinct. The final QM/MM PMF of 200 ps sampling time is also displayed in Figure 8 as the blue curve. The QM/MM PMF agrees with the MM PMF very well in the locations of the wells and barriers. Although S_cen_ still corresponded to the lowest free energy when the ion was in the pore (−4 > *z* > −16 Å), it was higher by ~2 kcal/mol in energy than the entrance, and S_ext_ is ~5 kcal/mol above S_cen_. The QM/MM PMF exhibited markedly higher barriers for Cl^−^ entering and exiting the pore (8–10 kcal/mol), and conversely a small (~0.5 kcal/mol) barrier from S_ext_ to S_cen_.

## Discussion

### External gating by Glu148

As revealed from our analysis, rotation of Glu148 side chain was necessary to make the pore entrance fully exposed to the Cl^−^ ion. The average χ2 values were 104°, 167°, and 78° for 0–1.8, 1.8–4.4, and 4.4–10 ns, respectively. The corresponding values are 53°, 64°, and 64° in χ1 and 179°, 154°, and 201° in χ3, respectively (note that χ3 has been offset to be in the range of 0–360°). The ~60° change in χ2 upon pore opening was much larger than the variations of ~10° in χ1 and ~25° in χ3. The first and third average values of χ2 (and of χ3 are similar, signifying the side chain returning to partially block the pore entrance after departure of the Cl^−^. We suspect that their existing small difference of ~20° was due to the presence of Cl^−^ at the entrance during the first 1.8 ns.

The pattern of dihedral evolution seems in line with what have been seen in the experimental structures of EcCLC. In the crystal structure of the wild-type (WT) protein, which is in the closed state, χ1-χ3 (averaged over chains A and B) are −25°, 70°, and −107°, respectively. For the E148Q mutant, which serves as a mimic of the open state, the average values of χ1-χ3 are −36°, −57°, and −84°, respectively. Thus, the changes are substantial (~130°) in χ2 but insignificant in χ1 (~10°) and χ3 (~20°). It is encouraging that both the computations and experiments support the gating role of Glu148 side chain.

Our observation of the side chain rotation as the external gating mechanism shares some similarities with what was found for a eukaryotic *Cyanidioschyzon merolae* CLC (CmCLC) transporter in a recent computational study (Cheng and Coalson, 2012). In that paper, constant-force steering was applied to guide Cl^−^ ions through the pore. No large-amplitude motions of the helixes were found. The side chain rotation of Glu210 (Glu148 equivalent in CmCLC) was characterized by significant changes in both χ1 and χ2 (>100°). In contrast, only changes in χ2 was notable in the present study.

We note that an earlier paper (Bisset et al., 2005) based on the simulations of a pure Cl^−^ channel CLC-0 suggested an alternative gating mechanism of Glu148: The Glu148 side chain was pushed back by the incoming Cl^−^ ions into a more central position and pressed against the channel wall, such that it did not block the travel of Cl^−^ ions. Such a conformation of the external gating residue was also detected in a recent experimental structure of CmCLC transporter (Feng et al., 2010). The discrepancies between this earlier and our current simulations may arise from the different proteins and/or the different starting geometries of Glu148 (the protonated Glu148 might stay initially deeper in the pore in the previous computation). On the other hand, it is certainly possible for the Glu148 side chain to move in either direction to escape from S_ext_. Therefore, while not observed in our simulations, we cannot rule out this alternative mechanism for pore opening. However, because EcCLC is an antiporter, the release of H^+^ to the extracellular side will probably require Glu148 be exposed to the extracellular solution sometime during a transport cycle.

### S_ext_-free vs. S_ext_-occupied open states

It is intriguing to see that the presence of the Cl^−^ ion in S_ext_ induced subtle local movements of Glu148 and Gly149 backbones. The approach of helix F to helix N can be attributed to the negatively charge of Cl^−^, which “glued” the two helix N-terminal ends. Note that these N-terminal ends are partially positively charged. The departure of the ion from S_ext_ thus widened S_ext_ again. These open-state geometries with and without the anion in S_ext_ can be labeled as S_ext_-free and S_ext_-occupied, respectively. Because the deprotonated carboxyl groups of Glu148 also carries a negative charge, the close-state geometry and the S_ext_-occupied open-state geometry should bear similarities in the S_ext_ region. This is indeed the case for the crystal structures (Dutzler et al., 2002, 2003). As can be seen in Table 1, the distances between Cα atom pairs for Arg147-Ala358, Glu148-Phe357, and Gly149-Ile356 (Figure 5B), which characterize the size of S_ext_, are rather similar across the three crystal structures. Averaged over the three pairs and over chains A and B, the distances are 7.0, 6.9, and 7.0 Å for WT, E148A, and E148Q, respectively. In view of this, the “true” open state could be the S_ext_-free one, for which E148Q without an anion at S_ext_ will be a realistic mimic.

**Table 1:** Average distances (in Å) between pairs of Cα atoms for selected residues: Arg147-Ala358, Glu148-Phe357, Gly149-Ile356, and Ser107-Tyr445.

		**Arg147-Ala358**	**Glu148-Phe357**	**Gly149-Ile356**	**Ser107-Tyr445**
SMD	0–2.0 ns	8.05	7.58	7.75	8.96
	2.0–4.4 ns	7.83	7.37	7.34	9.28
	4.4–10.0 ns	7.90	7.54	8.12	11.23
Exp	WT (chain A)	7.34	6.49	7.22	8.86
	WT (chain B)	7.38	6.53	7.24	8.84
	E148A (chain A)	7.06	6.22	7.50	8.65
	E148A (chain B)	7.08	6.06	7.62	8.70
	E148Q (chain A)	7.21	6.59	7.06	8.88
	E148Q (chain B)	7.28	6.53	7.11	8.90

*In addition to the SMD data, the experimental data are also provided for wild-type (WT, PDB code 1OTS) and two mutants E148A (PDB code 1OTT) and E148Q (PDB code 1OTU) (Dutzler et al., 2003)*.

We note that the distances in our SMD simulations are consistently larger than those from the crystal structure. Even for those of 2.0–4.4 ns corresponding to the S_ext_-occupied state, the distance average over these three pairs is 7.5 Å, ~0.5 Å wider than the crystal structures. The average distance over the S_ext_-free state is 7.8 Å. Currently we do not know exactly what caused the difference, although the different environments in which the protein stays may have contributed to the disparity—the protein is more tightly packed in the crystal form but probably more expanded when embedded in membrane.

### Quantum effects of electron delocalization on Cl^−^ translocation

Comparisons between the MM and QM/MM results obtained in this study provide an opportunity to gauge the quantum effects of electron delocalization on Cl^−^ transport by EcCLC. First, we point out that the MM (2 kcal/mol) and QM/MM (10 kcal/mol) barriers estimated here for Cl^−^ translocation are close to those (3–8 kcal/mol) obtained in previous computational studies (Cohen and Schulten, 2004; Gervasio et al., 2006; Ko and Jo, 2010a; Cheng and Coalson, 2012) again suggesting that the slow transport of Cl^−^ ions (~10^3^ s^−1^) by EcCLC (Walden et al., 2007) arises from other factors not considered here. The same locations of the barriers and wells in both MM and QM/MM PMFs implied that electron delocalization was fine tuning rather than dramatically changing the Cl^−^ transport mechanism. However, the QM/MM barriers are much higher than the MM barriers, suggesting stronger binding, especially at the entrance and S_cen_, at the QM/MM level than at the MM level.

**Figure 9:**
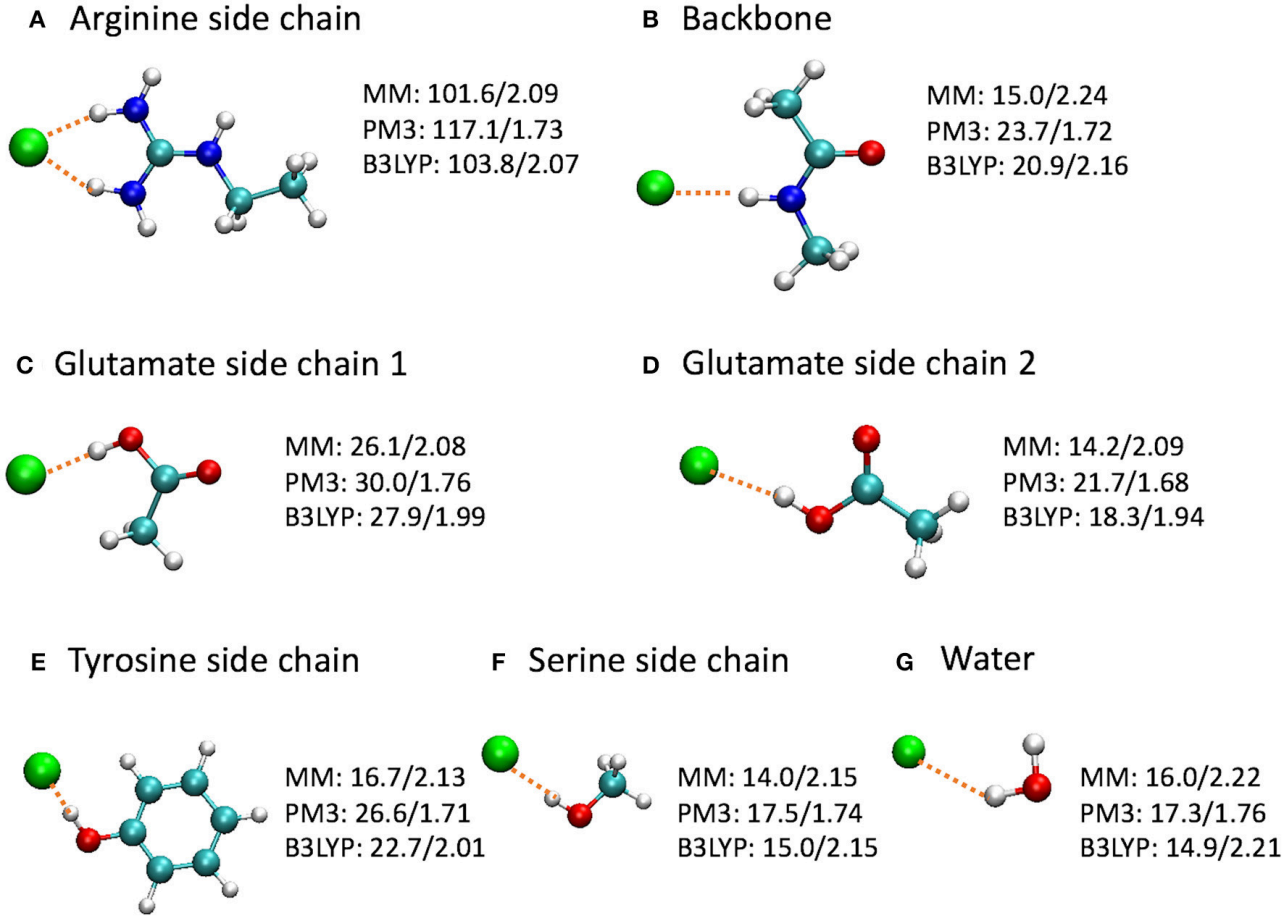
Optimized model complexes formed between Cl^−^ and **(A,C-F)** side chain, **(B)** backbone, and **(G)** water models, where the binding energies (kcal/mol)/H–Cl distances (Å) are listed next to each complex. The binding energy is defined as *E*(bind) = *E*(Cl^−^) + *E*(model) – *E*(complex), where the energies are at the optimized geometries at the given level of theory. The average H–Cl distance was shown for the arginine side chain model. The MM calculations employed the CHARMM36 force field and TIP3P water model.

To gain a deeper insight on the differences, we computed the binding energies for a few model complexes formed between Cl^−^ and residue backbone or side chains at the CHARMM36, PM3, and B3LYP/6-31+G(d) levels (Figure 9). Note that the Glu–Cl^−^ complex has two different conformations, both of which had been observed for Glu148 in the umbrella sampling trajectories. The positively charged arginine side chain was also included because of the strong attraction between the Arg147 side chain and the Cl^−^ in the entrance, although the Arg147 side chain is not part of the entrance cohort. Among the three levels of theory, the B3LYP calculations are the most accurate and serve as the reference for comparisons. Overall, the QM and MM optimized geometries were similar. However, in the PM3 calculations, the binding energies were overestimated by 7–17% and the bond lengths were shorter by 10–20% than the reference calculations. More specifically, the errors were 13 kcal/mol for the arginine side chain and 2–4 kcal/mol for the other models. In contrast, the H–Cl distances by CHARMM36 calculations agreed almost perfectly with B3LYP for water, serine side chain, and arginine side chain, but were somewhat too long (by ca. 4–8%) for the backbone and the side chains of glutamate and tyrosine. Not surprisingly, the CHARMM36 performance in energy seemed mixed. While performing remarkably well (within 2 kcal/mol, or within 7%) for water, serine side chain, and the first (*anti*) conformation of glutamate side chain, CHARMM36 noticeably underestimated the binding energies for the backbone, tyrosine side chain, and the second (*syn*) conformation of glutamate side chain by 4–6 kcal/mol, or 23–28%.

Interestingly, the model complexes on which CHARMM36 performed poorly contain π bonds. As our earlier study revealed, these π bonds stabilize the Cl^−^ ion substantially through mutual polarization and partial charge transfer (Church et al., 2013). The MM calculation failed to account for these quantum effects of electron delocalization in anion-π interactions. On the other hand, MM describes classical electrostatic interaction very well. Therefore, CHARMM36 significantly outperformed PM3 for the arginine side chain, which, although contains π bonds, interacts with Cl^−^ predominantly through electrostatic attractions. This also explains the different performances by MM for the two conformations of glutamate side chain. As the Cl^−^ ion stands closer to the CH_3_ group in the first conformation, electrostatic interactions contribute a larger share in the total binding energies. This was confirmed by natural orbital analysis, which revealed less electron delocalization in the first conformation than in the second: the Cl^−^ ion lost 0.107 e in the first conformation but 0.140 e in the second conformation. As expected, CHARMM36 reproduced the interaction energy of the first conformation more accurately.

One may argue that the MM parameters were optimized for condensed-phase simulations, while these model complexes are gas-phase calculations. This is certainly true. However, this means that MM typically *overestimates* the binding energies for gas-phase models. In contrast, MM underestimates the binding affinities here. Thus, these inaccurate MM energies are most likely caused by the missing stabilizations due to quantum electron delocalization.

While the overestimation of binding energies at the PM3 level likely increased QM/MM barriers, the underestimation of binding energy at the CHARMM36 level probably lowered MM barriers. In particular, the significantly overestimated attraction by Arg147 side chain near the entrance at the PM3 level contributed to an artificially deep well in 0 > *z* > 2 Å in the QM/MM PMF. The dip at S_cen_, however, might be described more realistically in the QM/MM PMF curve because of the substantial under-binding with the Tyr445 side chain by CHARMM36. It is a bit puzzling that S_ext_, where the Cl^−^ ion was extensively coordinated by backbone amine groups, was ~5 kcal/mol higher than S_cen_ in the QM/MM PMF, despite the over-binding with the backbone in the PM3 calculations. We do not know the reason, but it could be due to that the QM/MM PMF had not fully converged, as the barrier between S_ext_ and S_cen_ seemed to keep increasing with extending simulation time (Figure S8).

The differences between CHARMM36 and PM3 bindings also manifest in the pore-size plots (Figure 6). The stronger PM3 binding led to generally smaller pore sizes in QM/MM, especially in the binding sites. Interestingly, while the MM pore radii were ~1.8 Å or larger, which correspond to the generally accepted Cl^−^ ionic radius of 1.81 Å (Shannon, 1976) the QM/MM radii could go down to as small as ~ 1.4 Å. This smaller size indicated that the anion's size was effectively reduced as it lost substantial amount of its electron density. For reference, we note that the radius of the charge-neutral Cl atom was determined to be 0.99 Å (Pyykkö and Atsumi, 2009).

## Summary

We have carried out SMD simulations to escort a Cl^−^ ion through the pore of EcCLC to identify the accompanied local and global conformational changes of the protein. We observed that the side chain rotation of the external gating residue Glu148 was essential for the full access of the pore entrance by Cl^−^. Occupation of S_ext_ by the anion induced small but non-negligible shifting of the Glu148 and Gly149 backbones, which came closer to Cl^−^ for better ion solvation. This local conformational change implied subtle differences between the S_ext_-free and S_ext_-occupied open states. The ion's passing through the internal gating residue Tyr445 prompted helix R, which extrudes into the intracellular solution, to swing away from helix D. Helix R returned to its initial position once the ion exited sideways from the pore. There was substantial electron delocalization of Cl^−^ to its surroundings, especially to the π-bonds (e.g., the Tyr445 side chain), along the ion's journey through the channel. Umbrella sampling simulations at both the MM and QM/MM levels were performed to quantify the free-energy profile associated with Cl^−^ transport. The results identified major barriers for Cl^−^ moving into S_ext_ from the extracellular vestibule and exiting the pore from S_cen_. The barrier heights were ~2 kcal/mol in MM and ~10 kcal/mol in QM/MM, respectively. The higher barriers in the QM/MM than MM free-energy landscapes were attributed to the differences in the interaction energies between Cl^−^ and nearby residues, which were overestimated by PM3 but underestimated by CHARMM36. The QM/MM and MM results here probably provide the upper and lower bounds for the barrier heights. The weaker binding interactions predicted by MM was primarily caused by missing stabilization from electron delocalization between Cl^−^ and functional groups containing π bonds. The findings here suggest that quantum effects of electron delocalization may be more important than previously considered and should be taken into account if more accurate descriptions are desired for Cl^−^ transport proteins.

However, some of the above conclusions must be taken with caution, considering the intrinsic limitations in the model and methods employed this study. The current model system consisted of only one independent subunit of the transmembrane domain, while EcCLC contains a C-terminal domain and usually forms dimers. The amplitude of motion for helix R may be decreased in the presence of the C-terminal domain and the other subunit. The slowest steering speed in the SMD simulations was 2 Å/ns, which, although quite slow, might still be too fast for near-equilibrium steering. The QM/MM umbrella sampling was conducted employing the semi-empirical PM3 Hamiltonian with relatively short (200 ps per window) sampling time due to computational cost considerations. More advanced electronic-structure theory and longer simulations times are certainly desired for more accurate calculations. Classical polarizable force fields that explicitly include many-body contributions offer another choice (Kaminski et al., 2004; Patel and Brooks, 2004; Patel et al., 2004; Jorgensen, 2007; Warshel et al., 2007; Xie and Gao, 2007; Cisneros et al., 2014, 2016; Vanommeslaeghe and MacKerell, 2015; Albaugh et al., 2016). Although polarizable force fields are more expensive than standard non-polarizable force fields, they are more efficient than QM calculations. The disadvantage is that force fields based on classical electrostatics do not explicitly account for quantum effects such as polarization-exchange coupling and charge transfer (Illingworth and Domene, 2009). The simulations translocated only one Cl^−^ ion, leaving out the scenarios of two or more Cl^−^ ion moving together. The existence and movements of other Cl^−^ ions in the channel will likely impact the binding and translocation of a given Cl^−^ ion. The possible coupling between Cl^−^ ion transport and H^+^ delivery was also missed. Finally, the model we constructed was an “open-state” model, so the conversion between the closed and open states has not been visited. Future studies will need to address these issues.

## Author contributions

HL designed the project. AD carried out the code implementation. C-HW, BA, MZ, and AD performed the calculations. C-HW, AD, BA, and HL analyzed the results. C-HW, AD, and HL wrote the manuscript, which is then revised by all authors, who have given approval to the final version of the manuscript.

### Conflict of interest statement

The authors declare that the research was conducted in the absence of any commercial or financial relationships that could be construed as a potential conflict of interest.
