# Supplementary material for: Chloride Ion Transport by the E. coli CLC Cl−/H+ Antiporter: A Combined Quantum-Mechanical and Molecular-Mechanical Study
Source: Front Chem. 2018 Mar 13;6:62. doi: 10.3389/fchem.2018.00062 (PMC5859129; doi:10.3389/fchem.2018.00062)

Supplementary Material

Chloride Ion Transport by the *E. coli* CLC Cl^{-}/H^{+} Antiporter: A Combined Quantum-Mechanical and Molecular-Mechanical Study

Chun-Hung Wang, Adam W. Duster, Baris O. Aydintug, MacKenzie G. Zarecki, Hai Lin*

Department of Chemistry, University of Colorado Denver, Denver, CO, USA

*** Correspondence:**
Dr. Hai Lin
hai.lin@ucdenver.edu

Table of Contents

Figure S1 .... **3**
Figure S2 .... **4**
Figure S3 .... **5**
Figure S4 .... **6**
Figure S5 .... **7**
Figure S6 .... **8**
Figure S7 .... **9**
Figure S8 .... **10**

Figure S1. (A) Root-mean-square-deviation (RMSD) values of the protein backbone heavy atoms and **(B)** the *z* Cartesian coordinates of the Cl^- ion for steered molecular dynamics simulations. Steering progress is defined as t/t_{total}, where t is the progressing time and t_{total} the total time for each simulation (roughly 10, 4, and 2 ns for the steering speeds of 2, 5, and 10 Å/ns, respectively).

(A)

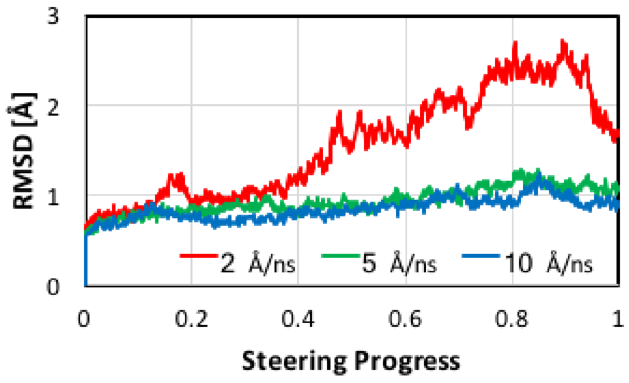

(B)

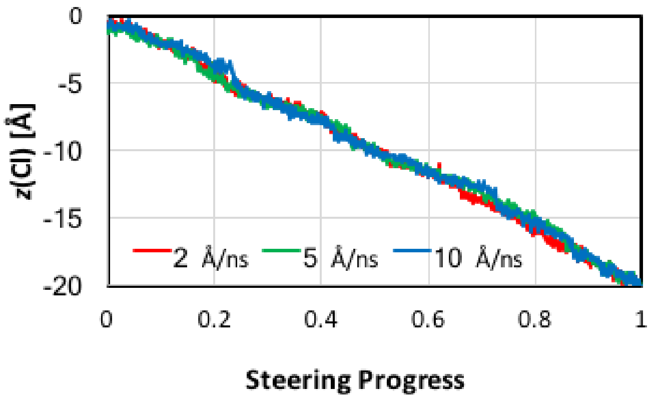

Figure S2. Root-mean-square-deviation (RMSD) values of the backbone heavy atoms of the helixes A to R as functions of the simulation time for the steering speed of 2 Å/ns.

(A)

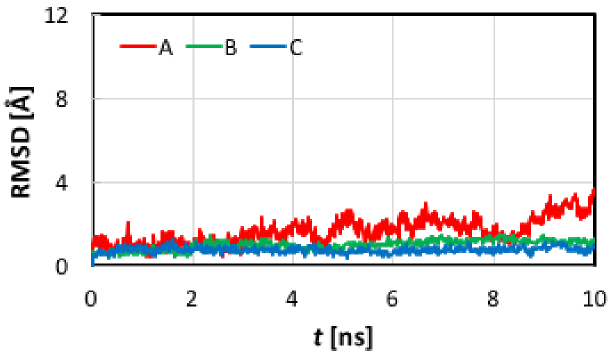

(B)

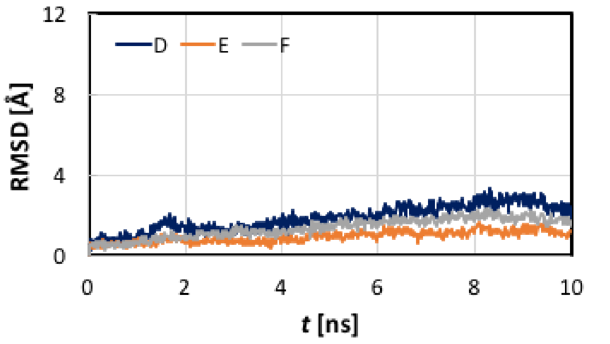

(C)

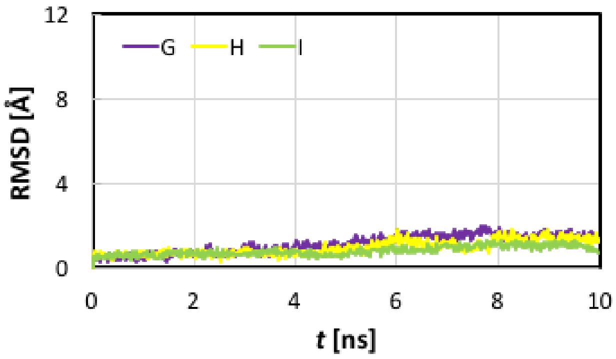

(D)

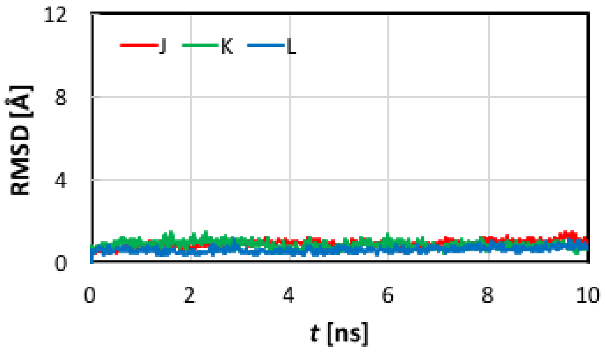

(E)

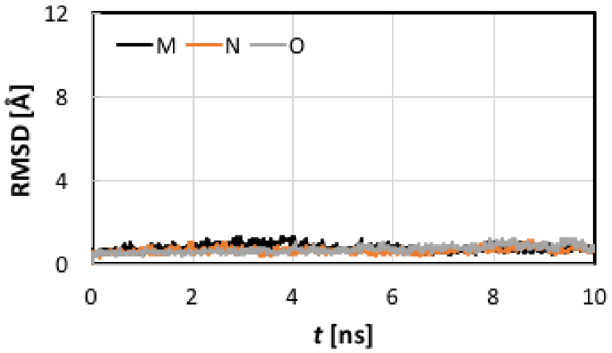

(F)

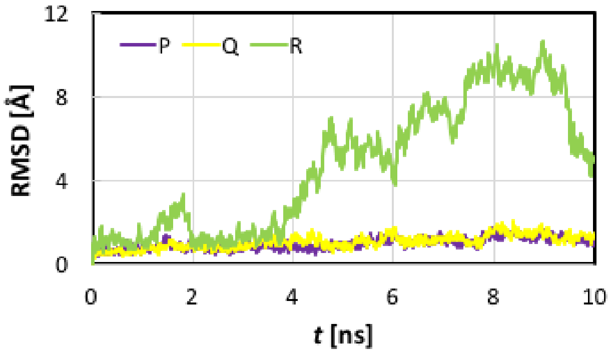

Figure S3. Comparisons of root-mean-square-deviation (RMSD) values of the backbone heavy atoms of helix A as functions of the simulation time for the steering speeds of **(A)** 2, **(B)** 5, and **(C)** 10 Å/ns.

(A)

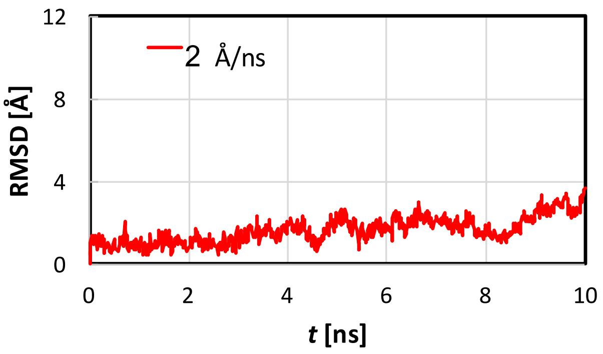

(B)

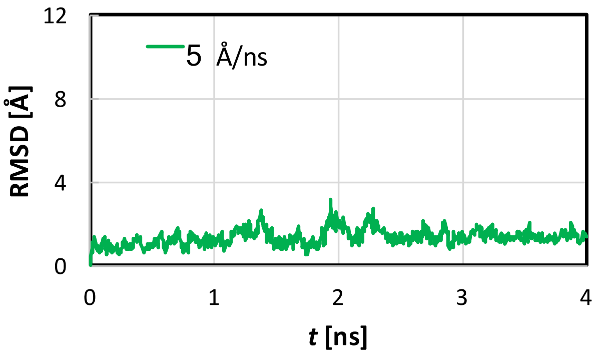

(C)

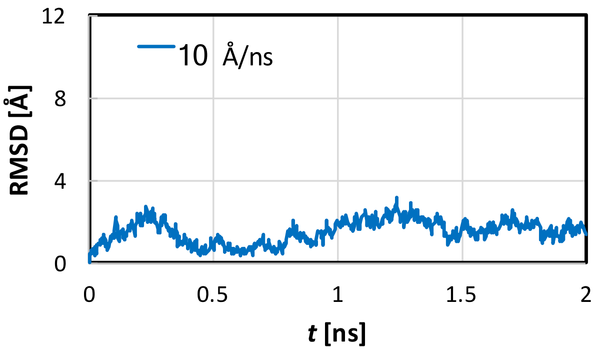

Figure S4. Root-mean-square-deviation (RMSD) values of the heavy atoms of the backbone (blue) and of both backbone and side chain (orange) for selected residues as functions of simulation time in the SMD simulations with a steering speed of 2 Å/ns.

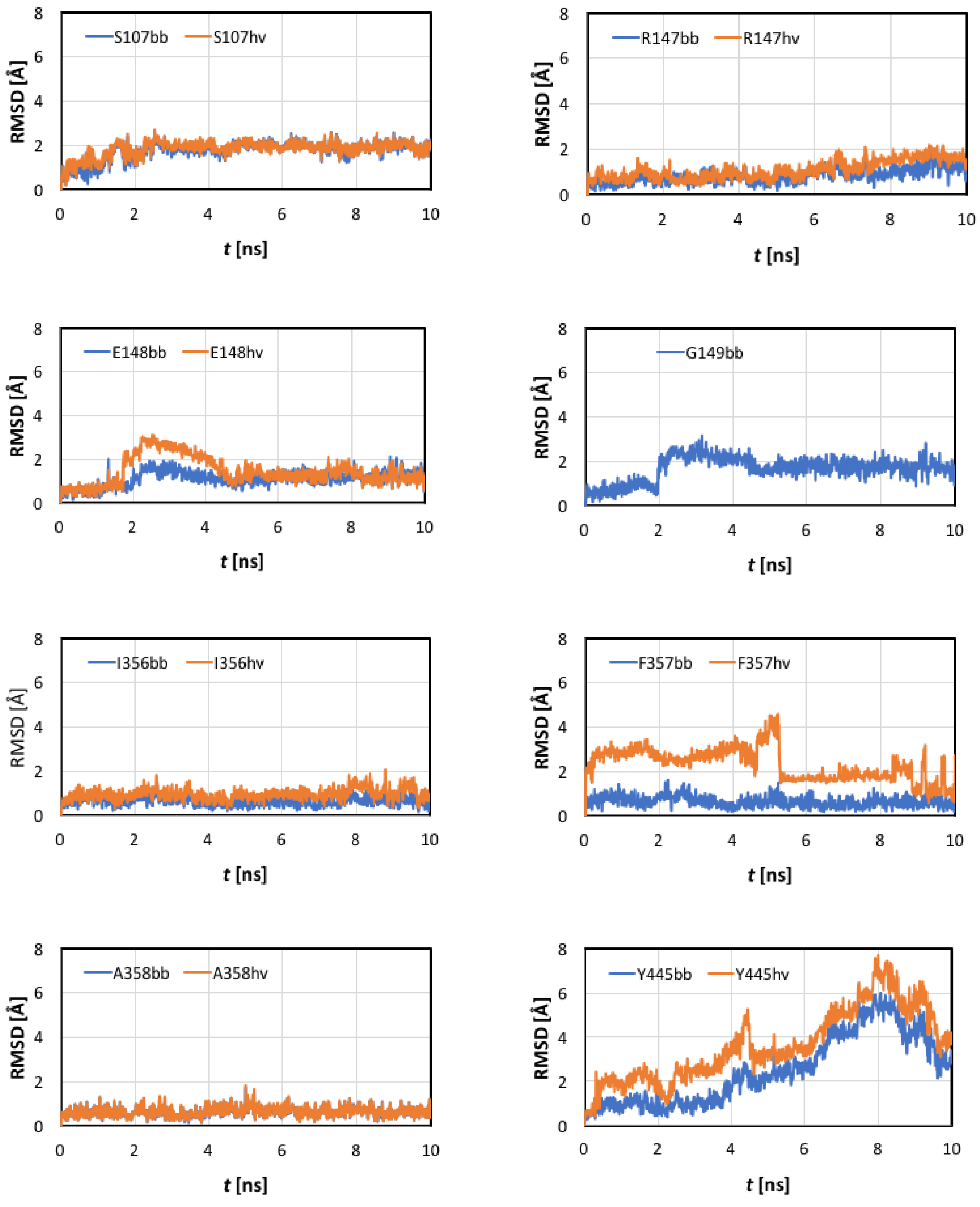

Figure S5. Distance between the transferred Cl^- ion and selected protein heavy atoms (left panel) or H atoms (right panel) as functions of the simulation time of the steering molecular dynamics simulations with a steering speed of 2 Å/ns. **(A)** The residues formed the first solvation shell of the ion before it entered the pore. **(B)** and **(C)** The same as (A), but for the residues when the ion toured through the S_{ext} site and after the ion left the S_{ext} site, respectively.

(A)

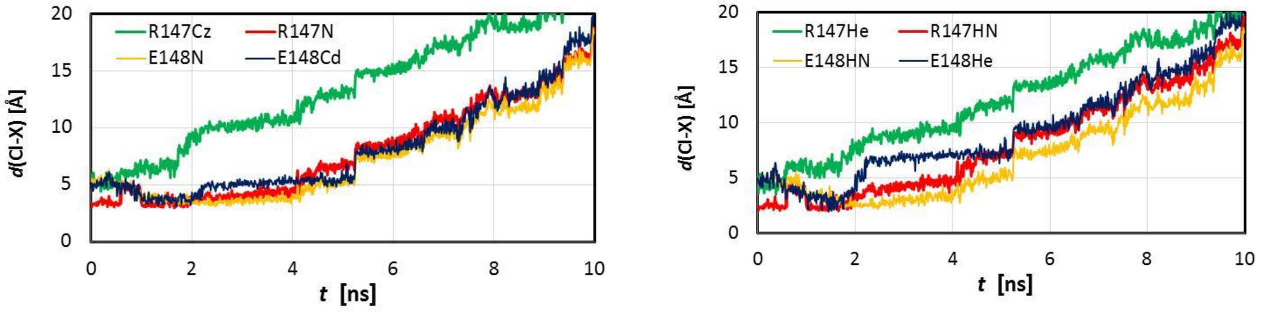

(B)

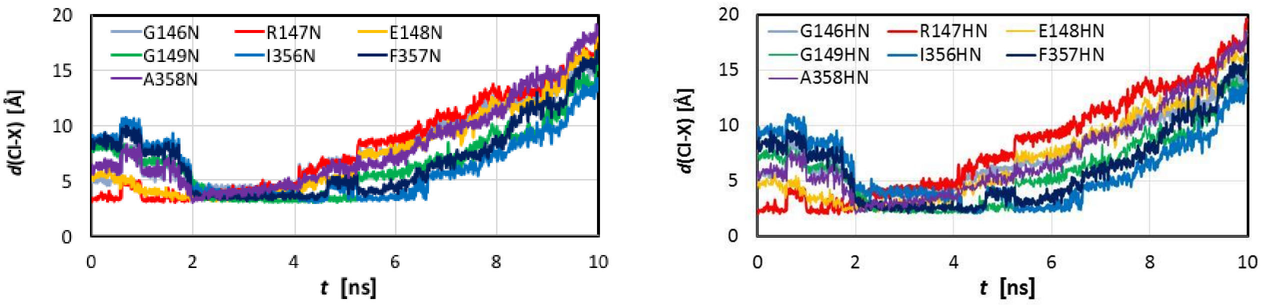

(C)

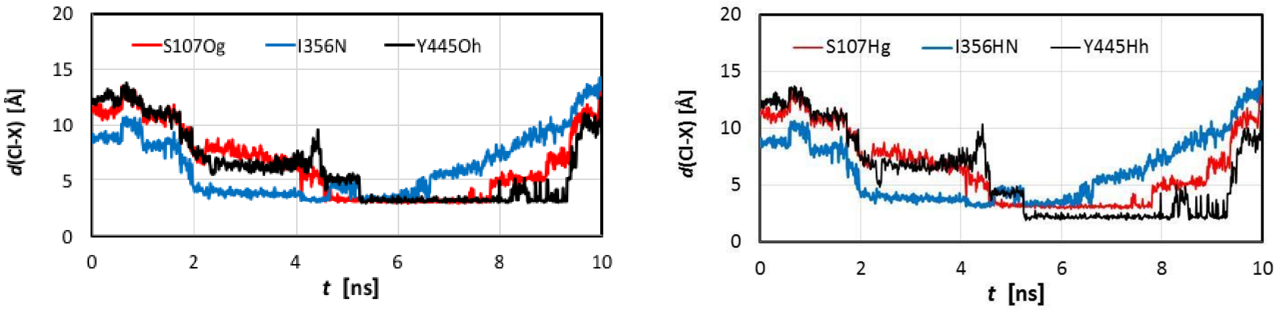

Figure S6. Side chain dihedrals of **(A)** Ser107 and **(B)** Tyr445 as functions of simulation time in the SMD simulations with a steering speed of 2 Å/ns. The torsional motion of the hydroxyl group was characterized by θ. Note that $\chi 1$ of Ser107 and θ of both residues have been shifted to be in the range of 0 to 360° instead of the standard –180° to +180°for better visualization.

(A)

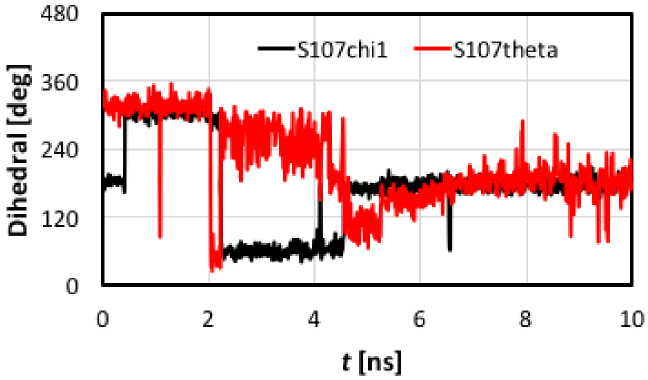

(B)

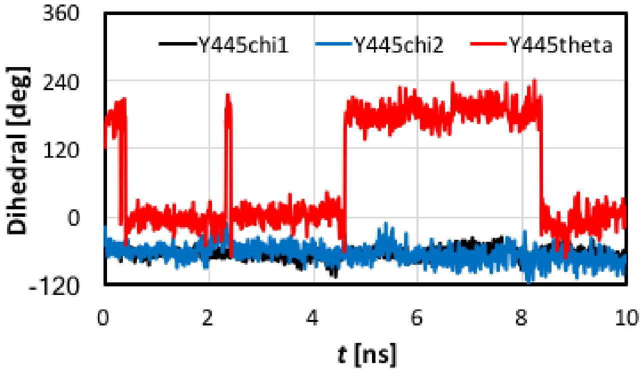

Figure S7. QM-computed **(A)** natural and **(B)** Löwdin charges of selected atoms or functional groups against the reaction coordinate *z* for representative geometries in QM/MM umbrella sampling simulations. For the protein residues, "sc" denotes side chain, "bb" denotes backbone, "(-COOH)" denotes side chain carboxyl group, "(-OH)" denotes the side chain hydroxyl group, "(ring)" denotes the ring in the tyrosine side chain, and "HN" denotes the backbone amine H. To assist better visualization, we have divided the reaction pathway into 10 sections (each of 2 Å long) and collected the average charges over each section.

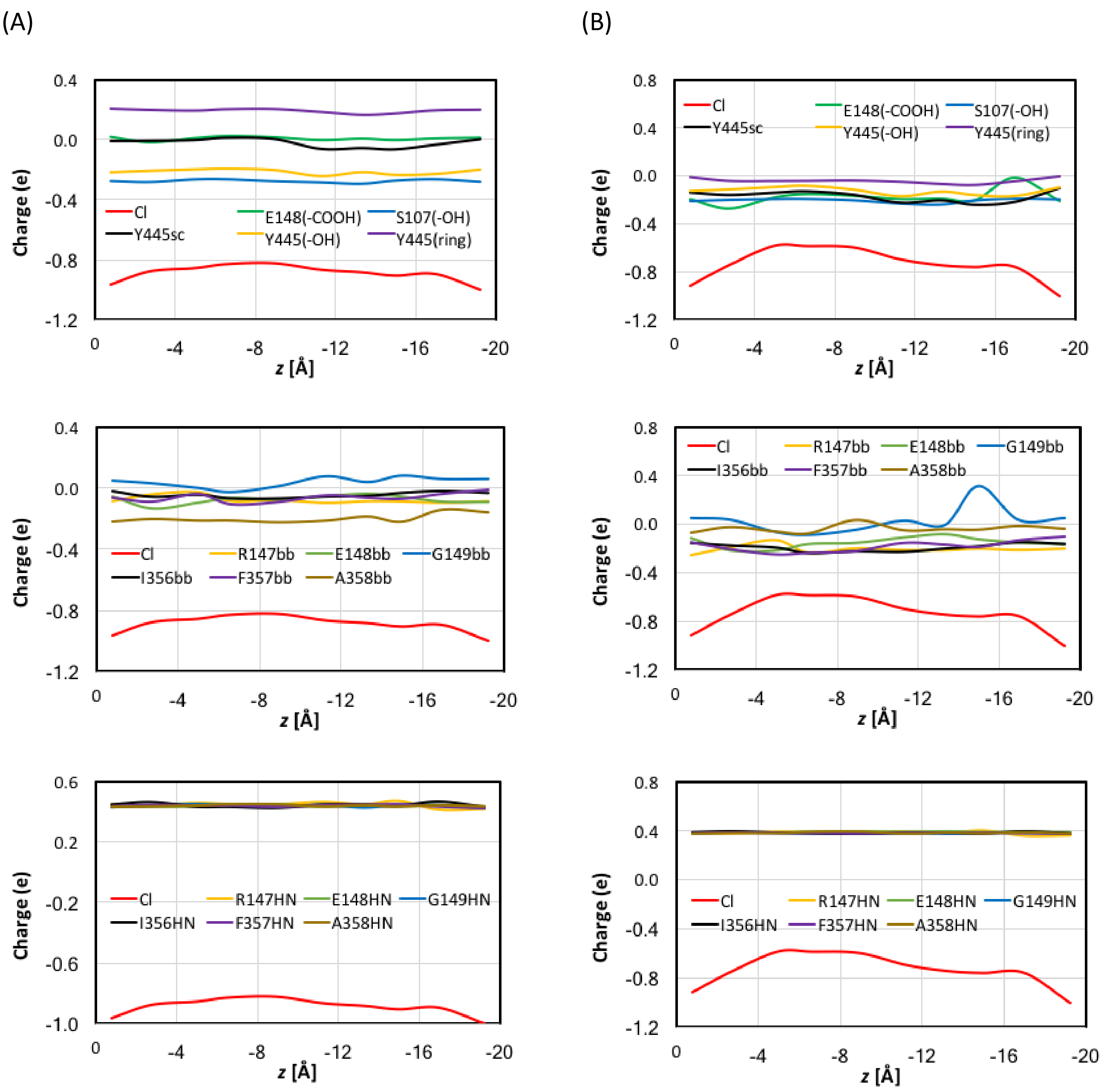

Figure S8. Potential of mean force (PMF) by **(A)** MM and **(B)** QM/MM umbrella sampling with different sampling times each window.

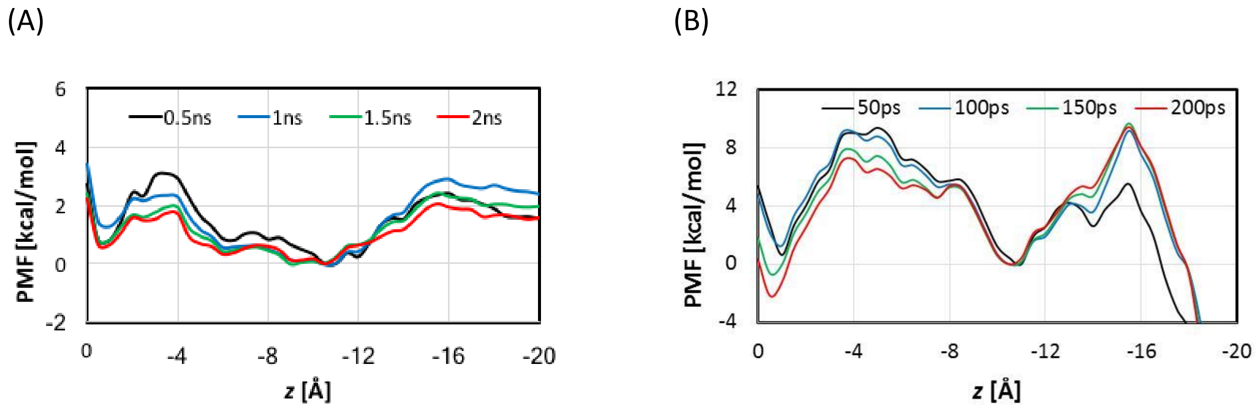

Supplement: Supplementary file 1 [file Presentation1.PDF]
